# Integrated microbiota and metabolite profiles link Crohn’s disease to sulfur metabolism

**DOI:** 10.1038/s41467-020-17956-1

**Published:** 2020-08-28

**Authors:** Amira Metwaly, Andreas Dunkel, Nadine Waldschmitt, Abilash Chakravarthy Durai Raj, Ilias Lagkouvardos, Ana Maria Corraliza, Aida Mayorgas, Margarita Martinez-Medina, Sinah Reiter, Michael Schloter, Thomas Hofmann, Matthieu Allez, Julian Panes, Azucena Salas, Dirk Haller

**Affiliations:** 1 Chair of Nutrition and Immunology, Technical University of Munich, Freising-Weihenstephan, Germany; 2 Leibniz-Institute for Food Systems Biology, Technical University of Munich, Freising, Germany; 3 Helmholtz Zentrum München, German Research Center for Environmental Health, Research Unit Comparative Microbiome Analysis, Munich, Germany; 4 ZIEL Institute for Food and Health, Technical University of Munich, Freising, Germany; 5 Inflammatory Bowel Disease Unit, Hospital Clínic de Barcelona, IDIBAPS, CIBERehd, Barcelona, Spain; 6 Laboratory of Molecular Microbiology, Department of Biology, Universitat de Girona, Girona, Spain; 7 Chair of Food Chemistry and Molecular Sensory Science, Technical University of Munich, Freising, Germany; 8 APHP, Hôpital Saint Louis, Department of Gastroenterology, INSERM UMRS 1160, Paris Diderot, Sorbonne Paris-Cité University, Paris, France

**Keywords:** Microbiology, Microbiology, Molecular biology, Molecular biology, Diseases

## Abstract

Gut microbial and metabolite alterations have been linked to the pathogenesis of inflammatory bowel diseases. Here we perform a multi-omics microbiome and metabolite analysis of a longitudinal cohort of Crohn’s disease patients undergoing autologous hematopoietic stem cell transplantation, and investigational therapy that induces drug free remission in a subset of patients. Via comparison of patients who responded and maintained remission, responded but experienced disease relapse and patients who did not respond to therapy, we identify shared functional signatures that correlate with disease activity despite the variability of gut microbiota profiles at taxonomic level. These signatures reflect the disease state when transferred to gnotobiotic mice. Taken together, the integration of microbiome and metabolite profiles from human cohort and mice improves the predictive modelling of disease outcome, and allows the identification of a network of bacteria-metabolite interactions involving sulfur metabolism as a key mechanism linked to disease activity in Crohn’s disease.

## Abstract (web summary)

Gut microbial and metabolite alterations are linked to inflammatory bowel diseases pathogenesis. Here the authors identify functional microbiota signatures that correlate with disease activity by comparing patients with Crohn’s disease undergoing autologous hematopoietic stem cell transplantation who either did not respond to therapy, experienced relapse after remission or maintained remission, and show that these microbial signatures recapitulate disease activity when transferred to mice.

## Introduction

Crohn’s disease (CD) is a chronic remitting and relapsing inflammatory disease of the gastrointestinal tract. Disease pathogenesis is suggested to be driven by complex interactions of genetic^1,2^, environmental, immune, and microbial factors^3^. This inherent complexity of the disease, manifested in a widely variable clinical course, makes it difficult to dissect disease mechanisms and to predict disease progression based on the patient’s status at initial diagnosis. In support of a pathogenic role of the gut microbiota in CD, numerous cross-sectional studies showed CD-associated dysbiosis to be characterized by reduced gut bacterial diversity, together with changes in relative abundance of certain taxa, such as *Fusobacterium*^4^, *Escherichia*^5,6^*, Faecalibacterium*, *Roseburia, Ruminococcaceae*, *Peptostreptococcaceae, Christensenellaceae*, and *Collinsella*^7,8^. As a first attempt to better understand the role of dysbiosis in CD, prospective cohorts are the best means to capture the changes that precede or follow disease onset and to link these shifts with mechanisms of disease pathogenesis^9^. Longitudinal studies with CD patients demonstrate dynamic fluctuations of the gut microbiome during disease^10^ and machine-learning algorithms identified microbial signatures associated with disease phenotypes, activity^7^, and response to therapy^11^. Recent efforts to integrate multi-omics analyses (i.e., metagenomics, metabolomics, transcriptomic, and proteomics) started to bridge the gap between bacterial community structure, their functional capacity and metabolic activity at the interface of microbe-host interactions^12^.

Metabolic activities of the microbiome play a central role in maintaining vital physiological processes of the host, including energy harvest^13^, protection against pathogens^14^, and modulation of host immunity^15^. Alterations in metabolite profiles are associated with functional changes in the microbiome and with the development of IBD^12^. First microbiome studies showed significant differences between IBD and healthy subjects based on metabolite profiles, and identified specific compounds to be correlating with disease state^16,17^. Additionally, a number of studies showed that the integration of clinical data is useful in building models that could accurately classify patients by response to therapy based on microbiome taxonomy and functional capacity^8,11,18^. Nevertheless, the causal role of dysfunctional gut microbiota in driving IBD flares of individual patients is poorly understood and requires the implementation of translational models^19^. Transplantation of fecal microbiota from patients into germ-free recipient mice has been used to recapitulate a variety of disease phenotypes, including IBD, and therefore provides a clinically relevant tool to mechanistically address microbe-host interactions^20–23^. In this study, we took advantage of autologous hematopoietic stem cell transplantation (HSCT), a therapeutic intervention that has significant and prolonged effects on a subset of severe and highly refractory CD patients, bringing them into drug-free remission, with a proportion of these patients relapsing over time. HSCT proved to be successful, potentially by erasing exaggerated immune responses against gut microbes. In this cohort, 76% of transplanted CD patients achieved drug-free remission 26 weeks post-transplant. Response to therapy was maintained in 50% of patients after 5 years post-transplant. In addition, the majority of treatment refractory patients regained responsiveness to subsequent IBD medication, suggesting that HSCT effectively changed disease-course progression of severe CD in a majority of patients^24,25^. In contrast to allogeneic HSCT, with intestinal graft-versus-host disease (GvHD) as a severe clinical complication^26,27^, the functional role of microbiome alterations on disease progression and therapeutic relevance in refractory CD after autologous HSCT remains completely unclear.

To functionally link changes in the fecal microbiome and metabolome with the clinical response of 29 CD patients after HSCT therapy, we adopted an integrative multi-omics approach together with experimental validation in humanized gnotobiotic mice. The use of this approach in the context of a phenotypically well-characterized HSCT patient cohort has enabled us to identify functional fingerprints associated with therapeutic failure or success during disease progression and to improve our understanding on the contribution of gut microbial dysbiosis to severe CD pathology.

## Results

### Individualized gut microbial variations in Crohn’s disease

**Fig. 1:**
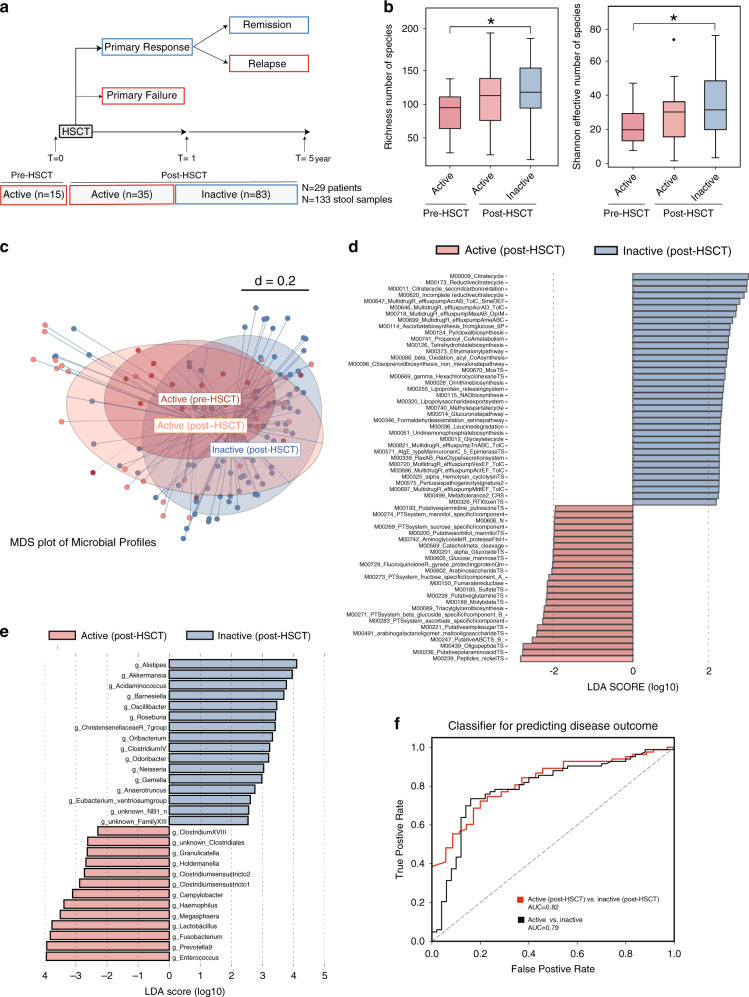
Individualized gut microbial variations in Crohn’s disease. **a** Overview of study cohort. We characterized 29 CD patients undergoing HSCT during 5-year follow up. Stool samples were collected both at baseline (pre-HSCT) and at various time points up to 5-year after HSCT (post-HSCT). Fecal samples collected at baseline (*n* = 15), during active state post-HSCT (*n* = 35) and during inactive state of disease (*n* = 83) are included in the analysis, **b** Alpha-diversity analysis measured by community species richness and diversity (Shannon effective number of species), showing reduced diversity in CD patients with active disease (before and after HSCT) compared to patient with inactive disease (after HSCT). For boxplots, 5–75% are shown. The upper whisker extends from the hinge to the largest value no further than 1.5 × IQR from the hinge (where IQR is the inter-quartile range, or distance between the first and third quartiles). The lower whisker extends from the hinge to the smallest value at most 1.5 × IQR of the hinge. Data beyond the end of the whiskers are outliers and are plotted individually. Significance is calculated by Mann–Whitney test. *p ≤ 0.05, **p ≤ 0.01, ***p ≤ 0.001. **c** MDS plot of microbial profiles of all stool samples stratified by time before and after HSCT and disease activity status (active pre-HSCT (*n* = 15), active post-HSCT (*n* = 35) and inactive post-HSCT (*n* = 83)). **d** Comparison of relative abundance of functional modules between CD patients with active or inactive disease (post-HSCT) using Linear discriminant analysis effect size (LEfSe) analysis generated from Phylogenetic Investigation of Communities by Reconstruction of Unobserved States (PICRUSt2). A number of 25 KEGG modules are significantly enriched in patients with active disease and 37 KEGG modules are enriched in patients with inactive disease. Data show differences in predicted bacterial metabolic function between CD patients with active or inactive disease (post-HSCT). Metabolic pathways enriched in active disease (red) and pathways enriched in patients with inactive disease (blue). **e** Comparison of relative abundance of bacterial genera between CD patients with active or inactive disease (post-HSCT) using LEfSe analysis. Taxa meeting an LDA significant threshold 2 are shown, active disease-enriched taxa (red) and taxa enriched in patients with inactive disease (blue). **f** Receiver operating characteristic (ROC) curves for all bacterial taxa to classify CD patients based on disease activity, or disease outcome post-HSCT. For each dataset, we ran random forest (RF) models to classify disease state and treatment response separately. OTU tables from each dataset were preprocessed and normalized as described in the Methods section. RF implemented in the WEKA software suite^61^ was used as a base-classifier and the number of trees was set to 100. The model was evaluated in a 10-fold cross-validation. HSCT Hematopoietic stem cell transplantation, LEfSe linear discriminant analysis effect size, PICRUSt2 Phylogenetic Investigation of Communities Reconstruction of Unobserved State. *p ≤ 0.05, **p ≤ 0.01, ***p ≤ 0.001.

First, we characterized fecal microbiota profiles of 29 CD patients undergoing HSCT at various time points up to 5 years post-transplant. Individual patient characteristics, clinical and endoscopic disease activity, and additional metadata are summarized (Table 1, Supplementary Tables S1–S3). We performed 16S rRNA gene sequencing on 133 fecal samples prospectively collected at baseline (*n* = 15) and post-HSCT including periods of active disease (*n* = 30 no-remission and *n* = 5 relapse) or inactive disease (*n* = 83 remission; Fig. 1a). Stratification of microbial profiles by disease activity showed reduced community richness and alpha diversity in CD patients with active disease (Fig. 1b). Beta-diversity analysis showed significant separation of microbial profiles between patients with active disease (pre-and post-HSCT) and patient with inactive disease (post-HSCT; Fig. 1c). To postulate putative functions of the microbial communities relevant to disease activity, we used Phylogenetic Investigation of Communities Reconstruction of Unobserved State (PICRUSt2) to infer the functional content of the microbiota based on 16S rRNA gene sequencing data. KEGG modules with significant differences in mean abundances (significance: log10FC > 2 and *p*-value < 0.01) in samples collected from patients with active or inactive disease were identified. (Fig. 1d). Looking at functional modules differentially abundant between patients identified metabolic pathways involved in sulfur transport system and other ion transport systems (e.g. Molybdate and Nickel) to be enriched in active disease (post-HSCT), while basic biosynthesis processes are enriched in inactive disease (post-HSCT; Fig. 1d). We next used discriminative LEfSe analysis (Linear discriminant analysis Effect Size) to identify differentially abundant genera. CD patients with active disease are enriched in members belonging to *Enterococcus*, *Fusobacterium*, *Haemophilus*, *Megasphaera*, *Campylobacter*, while *Roseburia*, *Christensenellaceae*, *Oscillibacter*, and *Odoribacter* are enriched in CD patients with inactive disease (Fig. 1e). Notably, using a 10-fold cross validated Random Forest classifier to predict different disease categories within the human cohort showed that the model can classify CD patients based on disease activity or based on clinical outcome post-HSCT with an area under the curve (AUC) = 0.79 and (AUC) = 0.82, respectively (Fig. 1f).

**Table 1:** Patients’ characteristics at inclusion.

	*n* = 29
Gender (male/female)	29 (8/21)
Age at diagnosis (years)^a^	19.24 ± 6.69
Disease behavior	
Inflammatory	19 (65.51%)
Fistulating	6 (20.68%)
Stenosing	4 (13.79%)
Smoking	
Active smoker	4 (13.78%)
Non-smoker	16 (55.17%)
Ex-smoker	9 (31.03%)
Disease location	
L1 (ileal)	1 (3.45%)
L2 (colonic)	7 (24.13%)
L3 (ileocolonic)	15 (51.72%)
L1 + L4 (ileal + upper disease)	1 (3.45%)
L3 + L4 (ileocolonic + upper disease)	5 (17.24%)
CDAI^a^	275.19 ± 94.81
SES-CD^a^	18.73 ± 8.78

*CDAI* Crohn’s disease activity index, *SES-CD* simple endoscopic score for Crohn’s disease.

^a^Mean ± SD.

^b^*n* (%).

To predict response to therapy based on microbial profiles of patients at baseline, we looked at a subset of patients (six responders and six non-responders) with stool samples collected at baseline and at week 26 post-HSCT, the primary endpoint for the assessment of HSCT responsiveness (Supplementary Fig. 1a). No significant differences in bacterial community structure or community richness and diversity were observed at baseline (Supplementary Fig. 1b, c). Generalized UniFrac distance analysis demonstrated large individual variations of bacterial communities before and after HSCT with no consistent pattern between patients (Supplementary Fig. 1d).

### CD fecal transplants reflect disease states in gnotobiotic mice

**Fig. 2:**
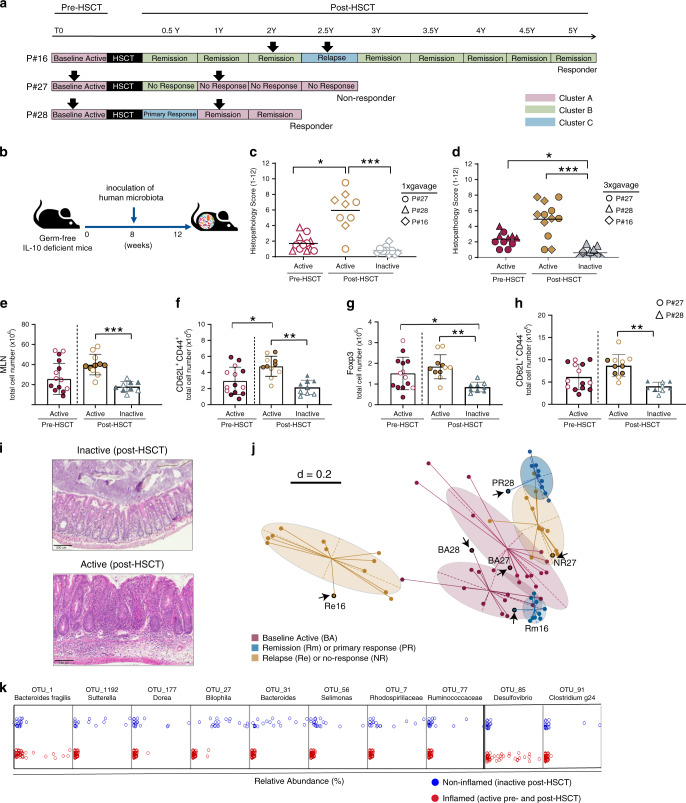
CD fecal transplant transfers disease states in gnotobiotic mice. **a** Disease course of three CD patients selected for humanization experiments (P#16, P#28, and P#27). Patients represented three different disease scenarios (relapse after long-term remission, sustained remission, and non-response). Fecal samples used for transfer into germ-free mice are indicated with arrows, they represent different time points during disease, different disease course and different microbial community configurations as indicated by Cluster type (A, B or C). **b** Experimental setup. GF WT (*n* = 65) and *Il-10*^*−/−*^ mice (*n* = 64) were colonized at the age of 8 weeks with selected CD patient-derived microbiota for 4 weeks by oral gavage one time or three times on 3 consecutive days as described in Methods section. **c** Histopathology of cecum tissue in ex-germfree *Il-10*^*−/−*^ mice colonized one time (open symbols) with microbiota from CD patients at baseline (*n* = 12), during active disease post-HSCT (*n* = 9) and during inactive disease post-HSCT (*n* = 10). **d** Histopathology of cecum tissue in ex-germfree *Il-10*^*−/−*^ mice colonized three times (closed symbols) with microbiota from CD patients at baseline (*n* = 11), during active disease post-HSCT (*n* = 12) and during inactive disease post-HSCT (*n* = 10). H&E staining was performed on tissues from all mice included in the analysis. One representative picture is included as an example. **e** Increased total count of CD4 T-cells in MLNs of mice humanized with samples from patients with active state of disease (P#27 and P#28). Absolute numbers of **f** central memory T cells (CD62LhighCD44high), **g** Treg cells (CD25+FOXP3+), **h** Naive T Cells (CD62Lhigh CD44low) among CD4 T-cell population. T cells gated in CD4+T cells were assessed by flow cytometry. Flow cytometry analysis was performed on mice colonized with microbiota from CD patients at baseline (*n* = 14), during active disease post-HSCT (*n* = 11) and during inactive disease post-HSCT (*n* = 9). **i** Representative H&E staining of cecum tissue in ex-germfree *Il-10*^*−/−*^ mice, colonized with microbiota from patients with active or inactive disease. **j** MDS of microbial profiles showing humanized mice (in halos) reflecting the individual features of their respective human donors (arrows). **k** 10-fold cross validated classifier identified 10 bacterial taxa differentiating humanized mice based on inflammation. The analysis was carried out in WEKA (Version 3.8). Random Forest implemented in the WEKA software suite^61^ was used as a base-classifier and the number of trees was set to 100. The model was evaluated in a 10-fold cross-validation. BA baseline active, PR primary responder, NR non-responder, Rm remission, Re relapse. Bars represent the mean ± SD. Significance for histopathology and flow cytometry data is calculated by Kruskal–Wallis one-way ANOVA followed by Dunn’s multiple comparison test. *p ≤ 0.05, **p < 0.01, ***p < 0.001.

To further address the functional impact of microbial communities on disease activity, we developed a humanized and IBD-relevant mouse model by colonizing germ-free *Il-10*^*−/−*^ mice with fecal samples from CD patients, as described in the Methods section. We used information on microbiota composition and stability in addition to disease activity/course and clinical response to HSCT of each individual patient to select representative human donor samples for functional validation in humanized mice. Using an unsupervised clustering approach, we identified three distinct clusters dominated by Bacteroidetes (Cluster A and B) or Firmicutes (Cluster C). Cluster B and C were also enriched in *Ruminococcaceae*, Alpha-, Gamma- or Delta- Proteobacteria (Supplementary Fig. 2a–c). We selected three-paired samples from CD patients representing different disease activities, disease course, community clusters, and overall bacterial community dissimilarity (Fig. 2a, Supplementary Fig. 2d, e). This included patient 16 who responded to HSCT, maintained remission for 2 years, and relapsed at month 29 post-HSCT; patient 28 who responded to HSCT and maintained remission for 2 years; and patient 27 who did not respond to HSCT therapy and maintained active disease (Supplementary Fig. 3a–c).

A total of 65 germ-free wild type (WT) and 64 *Il-10*^*−/−*^ mice at the age of 8 weeks were colonized via one-time or three-times oral gavage with selected CD patient-derived microbiota for 4 weeks (Fig. 2 b). Most importantly, the transplantation of microbiota from patients with active and inactive disease was enough to recreate the disease phenotype in all recipient *Il10*^*−/−*^ mice (active pre-HSCT vs. active post-HSCT, *p*-value = 0.0235, active post-HSCT vs. inactive post-HSCT, *p*-value < 0.0001; Fig. 2c). Boosting the bacterial load by inoculating mice three-times instead of one-time gavage did not change inflammatory responses in mice (active pre-HSCT vs. inactive post-HSCT, *p*-value = 0.0262, active post-HSCT vs. inactive post-HSCT, *p*-value < 0.0001; Fig. 2d). Tissue inflammation developed in the cecum as shown by histopathological evaluation (Fig. 2i). Wild-type mice remained disease-free, demonstrating the relevance of genetic susceptibility for disease progression (Supplementary Fig. 4a, b). *Il10*^*−/−*^ mice colonized with fecal microbiota from patients with active disease at baseline (pre-HSCT) developed milder inflammation compared to mice colonized with microbiota from patients with active disease post-HSCT (histopathology scoring: 1.7 ± 1.005 versus 5.9 ± 2.5). Remarkably, *Il10*^*−/−*^ mice colonized with fecal samples from patients in remission remained disease-free. T lymphocyte profiling in mesenteric lymph nodes (MLNs) of *Il10*^*−/−*^ mice colonized with fecal samples from a patient with active disease post-HSCT (P#27)) compared to mice colonized with fecal microbiota from a patient with inactive disease post-HSCT (P#28) showed significant higher frequencies of CD4+ central memory T cells (CD62LhighCD44high), Treg cells (CD25+FOXP3+) and naive T-Cells (CD62Lhigh CD44low; Fig. 2e–h, Supplementary Fig. 4c, d). Changes in immune cell profiles mirrored the level of tissue pathology and confirmed the transmissibility of disease activity in gnotobiotic mice. The strongest immune activation was observed in humanized mice colonized with microbiota associated with active disease (post-HSCT). 16S rRNA gene profiling showed that humanized mice reflected the dysbiotic features of their respective donors. Beta-diversity analysis clearly demonstrated that the community profiles of the recipient mice mimic the microbiota composition of the corresponding donor samples in six distinct community clusters (Fig. 2j). In addition, humanized mice and corresponding human donors showed similar phyla composition (Supplementary Fig. 3d) as well as community richness and diversity (Supplementary Fig. 3e), supporting the translational validity of the gnotobiotic mouse models. Nevertheless, humanized mice appeared to selectively enrich for individual-specific repertoires of OTUs differentially growing in higher abundance (Supplementary Fig. 3f). Using a machine-learning algorithm, we identified a signature of 10 taxa that discriminate humanized mice according to the inflammatory status. A signature characterized by increased relative abundance of *Bacteroides fragilis* and *Desulfovibrio* classified humanized mice according to inflammation with high accuracy (Fig. 2k).

### Multi-omics integration selects key targets of inflammation

To characterize the functional consequences of altered microbial compositions in CD patients during active disease post-HSCT, we performed UHPLC-TOF-MS metabolite profiling using reversed-phase ultra-high-performance liquid chromatography (RP-UHPLC) and hydrophilic interaction ultra-high-performance liquid chromatography (HILIC-UHPLC). In combination, these techniques cover polar and nonpolar metabolites and spectral features were detected. The number of features annotated after preprocessing, peak picking, and removal of near-zero variance features is summarized (Supplementary Table 4).

**Fig. 3:**
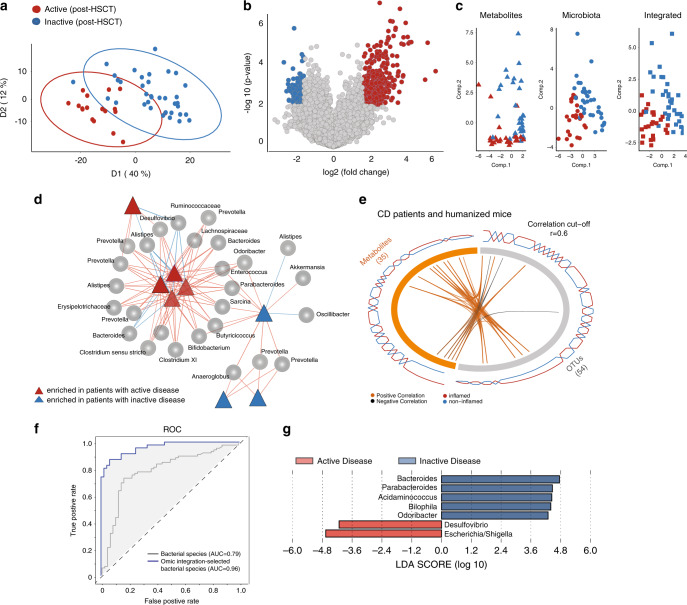
Multi-omics integration selects predictive targets of inflammation. **a** PLS-DA plot showing separation of metabolite profiles between CD patients with active (Red, *n* = 18) or inactive (Blue, *n* = 36) disease post-HSCT. **b** Volcano plot of differentially abundant metabolites selected based on fold change (log 2 ≥ 3) and significance (*p* < 0.01). **c** Separation of CD patients according to disease activity post-HSCT based on metabolite features, microbiota, and on the integrated microbiome-metabolome dataset**. d** Relevance network constructed from the similarity matrix to identify the association between OTUs (circles) and metabolite features (triangles), and their link to inflammation. Based on the inferred correlation matrix, variables exceeding a correlation coefficient threshold of (*r* = 0.7) are considered associated. Two clusters were identified based on the metabolites link to inflammation (red = enriched in CD patients with active disease, blue = enriched in CD patients with inactive disease). **e** Circos plot on the merged CD patients and humanized mice datasets showing the positive and negative correlation (*r* ≥ 0.6) between variables of different omics-data (OTUs and metabolites) and is built based on a similarity matrix. Individual tiles on the ring represent individual features, while the connecting lines (orange or black) in the center show positive or negative correlations within and across metabolomics platform above a correlation cut-off coefficient of 0.6. The additional line plots on the outer ring visualize the relative abundance of each feature per experimental group. **f** Receiver operating characteristic (ROC) curves of OTUs interacting with metabolites in the integrated analysis. **g** Linear discriminant analysis effect size (LEfSe) indicating differentially enriched bacterial OTUs at the genus level of human and mouse fecal bacterial communities corresponding to active disease (red) and inactive disease (blue).

Partial least squares projection to latent structures analysis (PLS-DA) clearly separated patients with active and inactive disease (Fig. 3a). Identification of differential metabolite features showed 332 and 119 differentially abundant features in patients with active or inactive disease, respectively (Fig. 3b). Metabolite identification using structural ontologies showed a trend towards regulation of sulfur metabolism pathways (Supplementary Fig. 5a, Supplementary Data 1), however enrichment analysis did not reach statistical significance. Supportive to the idea that multi-omics data improve discriminative analysis, the integration of microbiota and metabolite profiles of CD patients revealed a better separation based on disease activity compared to microbiota or metabolites only (Fig. 3c). To identify relevant microbiota-metabolite interactions, we performed correlation analysis on the differentially abundant features from both datasets, using canonical correlation analysis and partial least squares regression, as described before^28^ (Fig. 3e). A relevance network analysis highlighted two clusters, both including features from microbiota and metabolome datasets (Fig. 3d). We identified two hubs of OTU-metabolite interactions enriched with active or inactive disease states. While OTUs corresponding to *Enterococcus*, *Desulfovibrio*, *Prevotella*, *Alistipes*, *Ruminococcaceae*, *Lachnospiraceae* correlated with active disease, an enrichment of *Akkermansia*, *Oscillibacter*, *Anaeroglobus*, and *Prevotella* was associated with inactive disease. We next set out to identify bacterial taxa associated with metabolic changes in both CD patients and corresponding gnotobiotic mice. Although CD patients and respective humanized mice appeared to cluster separately (Supplementary Fig. 5c), the integrated microbiota-metabolome dataset allowed a clear separation between active and inactive disease (Supplementary Fig. 5d). Using correlation analysis as described before, we identified a subset of metabolites and OTUs in the combined and integrated dataset (Fig. 3e). A full list of interacting OTU and metabolite features is summarized (Supplementary Data 2). Classification of samples based on these selected microbiota features significantly improved the predictive modeling compared to what we have identified previously solely based on taxonomic composition (Fig. 3f). LEfSe analysis on the OTUs involved in these interactions, identified 7 OTUs to be differentially abundant between microbial communities corresponding to active or inactive disease. Intriguingly, OTUs corresponding to *Desulfovibrio* and *Escherichia/Shigella* were enriched in active disease, while OTUs corresponding to *Bacteroides*, *Parabacteroides*, *Bilophila*, *Acidamicococcus*, and *Odoribacter* were enriched in inactive disease (Fig. 3g).

### Sulfur metabolism links disease activity to human microbiome in humanized mice

**Fig. 4:**
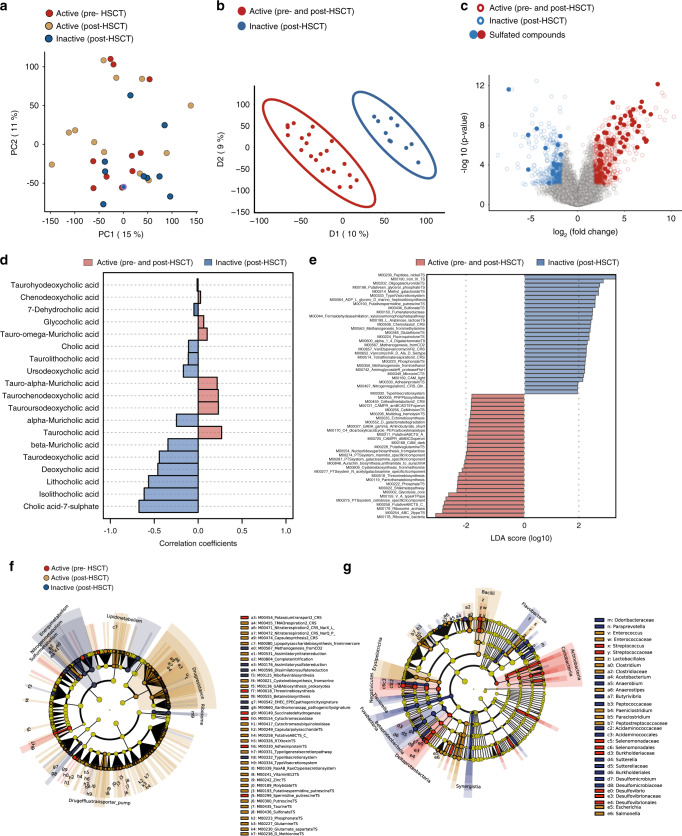
Sulfur metabolism links disease activity to human microbiome in humanized mice. **a** PCA score plot of metabolite profiles of mice colonized with microbiota from human donors with active disease pre-HSCT (*n* = 10), active (*n* = 12) or inactive (*n* = 10) disease post-HSCT. **b** PLS-DA plot comparison of metabolite profiles of mice based on disease activity of their original human donors (active pre-and post-HSCT) and (inactive post-HSCT). **c** Volcano plot of 1252 differentially abundant metabolites selected based on fold change log 2 ≥ 3) and significance (*p* < 0.01). **d** Correlation plot of bile acid concentrations and disease activity. **e** LEfSe generated from PICRUSt2 identified the most differentially abundant functional modules between mice colonized with microbiota from human donors with active (*n* = 37) or inactive disease (*n* = 18). A number of 42 KEGG modules significantly enriched in mice colonized with microbiota from patients during active disease and 42 KEGG modules were enriched patients with inactive disease (significance: log10FC > 2 and *p* < 0.01.). Inactive (post-HSCT) functional modules are indicated with a positive LDA score (blue) and modules enriched in (active post-HSCT) with a negative score (red). Only functional modules meeting an LDA significant threshold of >2 are shown. **f** Cladogram obtained from LEfSe analysis of functional genes according to KEGG using shotgun metagenomics. **g** Cladogram plotted from LEfSe analysis showing the taxonomic levels represented by rings with phyla in the outermost the ring and genera in the innermost ring. Each circle is a member within that level. Those taxa in each level are colored by disease state of human donor for which it is more abundant (*p* < 0.05; LDA score 2). KEGG Kyoto Encyclopedia of Genes and Genomes, LEfSe linear discriminant analysis effect size, PICRUSt Phylogenetic Investigation of Communities Reconstruction of Unobserved State.

Fecal transplantation of CD patients into GF mice selectively induced inflammation in humanized mice driven by a variety of gut community profiles, suggesting that different microbiota configurations infer similar functional activity in a susceptible host. We therefore characterized the metabolic alterations driven by inflammation in humanized mice and performed untargeted UPLC/TOF-MS metabolomics for mouse gut microbiome. An unsupervised principal component analysis (PCA) was performed to visualize the distribution of mouse metabolite profiles, based on the disease activity of the human donor (Fig. 4a). Differences across inflamed and non-inflamed mice were visualized by a PLS-DA analysis to identify metabolites that classify samples based on the presence of host inflammation (Fig. 4b). To select discriminating metabolites, a volcano plot was built based on the PLS-DA model (Fig. 4c). Of the captured 28,622 features, 672 features were differentially abundant in inflamed mice, while 580 features were differentially abundant in non-inflamed mice (Supplementary Table 4). Consistent to what we observed in CD patients, dysregulation of sulfur metabolism was linked to development of inflammation in humanized mice. A relatively high number of sulfated compounds, including bile acids, polyphenols and biogenic amines contributed to the differentially abundant metabolites (Supplementary Data 3). We next validated bile acid alterations of 19 primary, secondary as well as conjugated bile acids in human donors and their corresponding humanized mice using a targeted metabolomic approach. Correlation and fold changes analysis identified cholic acid 7-sulfate, lithocholic acid and its derivatives to be correlating with an inflammation-free state in mice (Fig. 4d). Conversely, tauro-conjugated bile acids and glycocholic acid correlated with inflammation, emphasizing previous studies reporting an increased levels of conjugated and sulfated bile acids in the feces of CD patients^16,29^. Putative functions prediction using PICRUSt2 showed an enrichment of functional pathways involved in sulfate or glutathione transport systems in non-inflamed mice. Conversely, inflamed humanized mice showed an enrichment of multiple amino acids (cysteine and threonine) biosynthesis, functional pathways involved in glycolysis, Type III secretion system and cell division transport system (Fig. 4e). To confirm these postulations, we performed shotgun metagenomics sequencing on the 6 microbial communities from the 3 human donors that have been transplanted into germ-free mice and 18 microbial communities from the corresponding humanized mice. Human donors were characterized by individualized functional profiles with almost no shared genes (Supplementary Fig. 6A). Remarkably, functional analysis of the gut microbiome of humanized mice clearly demonstrated that genes responsible for regulating the anabolism or catabolism of sulfur-containing amino acids (cysteine and methionine) significantly contributed to the separation between mice colonized with microbiota from CD patients with active or inactive disease (Fig. 4f). In mice colonized with microbiota from CD patients with inactive disease, we observed an enrichment of functional modules of glutathione biosynthesis, known to be involved in bio-reductive reactions against reactive oxygen species and xenobiotics. Notably, we observed increased abundance of modules involved in iron and nickel transport systems in mice colonized with inflammatory microbiota. Additionally, the enrichment of taurine and sulfonate transport systems (in inflamed mice) suggests an alteration of bile acid metabolism. Intriguingly, analysis of functional modules involved in sulfur metabolism using shotgun metagenomics data identified an enrichment of sulfonate, methionine, cysteine and taurine transport systems in mice colonized with microbiota from patients with active disease. In contrast, an enrichment of functional modules involved in assimilatory and dissimilatory sulfate reduction was observed in mice colonized with microbiota from patients with inactive disease (Supplementary Fig. 6b, c). The differential abundance of certain bacterial groups at family and genus levels is shown in the Cladogram (Fig. 4g). Members belonging to *Desulfovibrio, Enterococcus, Streptococcus*, and *Escherichia*are highly enriched in inflamed mice, while Burkholderiales, Desulfomicrobiaceae, *Sutterella*, and *Butyrivibrio* are highly enriched in non-inflamed mice. These metabolic changes are in line with the increased relative abundance of sulfate-reducing bacteria, including *Desulfovibrio* and *Clostridia* both of which use sulfate as a terminal electron acceptor for respiration and concomitantly produce hydrogen disulfide, a toxic metabolic byproduct.

## Discussion

In the present study, we investigated perturbations of community structure and metabolic activity in the gut microbiome of CD patients across longitudinal follow-up sampling after HSCT. This single-center cohort from Barcelona included 29 patients with severely active CD, being unresponsive and refractory to all conventional therapies. Six months after autologous HSCT, 76% of the recruited CD patients achieved drug-free remission. Around 15% of the patients maintained drug-free remission at 5-year follow up^24^. Despite its therapeutic efficacy, HSCT is an intervention with a relatively high mortality rate (2–10%)^30^, requiring a thorough and timely monitoring to identify patients who are most likely to respond to HSCT therapy.

We collected fecal samples at three main time points, at baseline, 26 weeks and 52 weeks post-transplant. Additional fecal samples were collected at various time points, depending on availability and clinical rational. Since the individual disease course also varied in this highly fragile cohort of patients, we generally performed a cross-sectional analysis based on clinical endpoints post-HSCT. Patients with active and inactive disease showed distinct microbial profiles and patients at baseline or during relapse showed overlapping microbial communities characterized by reduced community richness and diversity. Longitudinal sampling of CD patients over a 5-year period highlighted the fluctuations of microbial profiles during the disease course, and particularly, changes of disease status correlated with dramatic shifts in microbial community structure. These findings are in agreement with previous reports demonstrating that microbiome changes overtime underline the severity of inflammation^8,10,12^. Differentially abundant bacterial groups were previously suggested to be relevant in IBD^7,8,31–33^, and included members belonging to sulfate-reducing gamma- and deltaproteobacteria, butyrate-producing Clostridiales*, Enterococcus, Megasphaera, Campylobacter*, and *Fusobacterium*. Conversely, patients with inactive disease had increased relative abundance of beneficial microbes including *Akkermansia, Barnesiella*, *Oscillibacter, Roseburia*, *and Odoribacter*. Given the inter-individual variability and heterogeneity of the human gut microbiota, taxonomic composition was not enough to predict the therapeutic response to HSCT. We approached this challenge by applying multi-omics analysis comprising metagenomics profiling, in addition to targeted and untargeted metabolomics profiling. In addition, we assessed the functional potential and metabolic activity of gut microbial communities in gnotobiotic humanized mice. Appreciating the known limitation of incomplete human bacterial transfer into germ-free mice^34^, we captured key features of the patient-related dysbiotic microbiota, including a similar community structure and diversity, and robustly transferred the different disease states into the susceptible host. Going beyond microbial community structure, the quantification of vast numbers of metabolites in an untargeted fashion revealed shared functional metabolic pathways in inflammation involved in sulfur metabolism, bacterial toxins secretion systems, and purine metabolism. Previous findings suggested that hydrogen sulfide can be generated as a metabolic by-product of sulfate-reducing bacteria (e.g. *Desulfovibrio*). In turn, hydrogen sulfide inhibits Acyl-COA dehydrogenase; an enzyme required for butyrate oxidation which ultimately leads to impairment of butyrate oxidation, and disruption of the gut homeostasis^35–37^. Nevertheless, these compounds were not significantly enriched in our primary analysis, possibly due to the vast inter-personal variations between CD patients. However, humanized mice showed a significant enrichment of sulfated compounds under inflamed conditions, supporting the relevance of sulfur metabolism in the pathogenesis of CD.

While a potential detrimental role of H_2_S has been linked to IBD, particularly in UC^38,39^, much less is known about gut microbiota contributions towards sulfur metabolism and its role in intestinal inflammation. Intriguingly, our data showed disease-associated enrichment of sulfate-reducing bacteria in CD patients with active disease. Likewise, *Desulfovibrio, Bilophila*, *and Bacteroides fragilis*, all of which are involved in bacterial sulfur metabolism via different pathways, were among those bacterial groups discriminating humanized mice based on inflammation. Additionally, we observed an increased abundance of functional modules in transition metals transport systems (iron, nickel, and molybdate). These transition metals are known to serve as enzymatic cofactors contributing to the virulence of many pathogenic bacteria under inflammatory conditions^40^. Along the same line, we previously showed that deprivation of dietary iron prevented intestinal inflammation in *TNF*^*ΔARE/WT*^ mice, a mouse model of chronic ileitis^41^. Interestingly, intravenous iron replacement therapy in IBD patients circumvented the detrimental effect of oral iron supplements and had less impact on microbiota and metabolite profiles^42^. Pairing metabolite profiling and functional profiling using shotgun metagenomics confirmed a dysregulation of metabolic pathways involved in different aspects of sulfur metabolism. In this context, our data suggest that the generation of hydrogen sulfide seems to take place through other metabolic pathways. These included assimilatory and dissimilatory sulfate reduction pathways, sulfonate, and taurine transport systems as well as pathways involved in biosynthesis of sulfur-containing amino acids (e.g. cysteine and methionine), supporting the hypothesis that microbiota alterations towards higher SRB abundance increase detrimental effects of sulfur-containing metabolites and susceptibility to active CD. Colonizing germ-free mice with fecal microbiota from CD patients with different disease activity demonstrated that different microbial community structures can drive disease in a susceptible host, highlighting the relevance of the functional impact of gut microbiota beyond taxonomic classification. The degree of inflammation in humanized mice correlated with the patients’ disease severity and was reflected by the activation of detrimental immune responses. Prior research demonstrated an enhanced colitis severity and immune activation in ex-germ-free mice colonized with gut microbiota from IBD patients compared to healthy subjects^23^. Here, we showed that gut microbiota from CD patients in active disease (post-HSCT) induced a higher level of immune activation and an enhanced inflammation compared to gut microbiota from the same patients during remission or at baseline. The integration of microbiome-metabolome profiles from human and humanized mice improved predictive modeling of disease outcome. Since temporal and inter-individual variation of CD patients substantially contribute to the heterogeneity of data, consistent findings in humanized mice allowed better identification of disease-relevant functional signatures. An enrichment of sulfated compounds, including sulfated bile acids, reached significance in humanized mice associated with microbiota from patients with active disease. Targeted measurement of bile acids identified tauro-conjugated bile acids under inflammatory conditions, suggesting that bile acid deconjugation and production of H_2_S potentially leads to detrimental effects in the host. The uptake of taurine is achieved by taurine-specific transport systems, which are for example encoded in *Escherichia coli*^43^ and *Bilophila wadsworthia*^44^. The predominance of sulfated bile acids in inflammation is in accordance with previous reports showing the role of bile acid sulfation in the elimination and detoxification of bile acids^29,45^.

In conclusion, this study aimed to investigate and characterize functional signatures associated with microbiota changes in CD patients after HSCT, providing proof-of-concept that in spite of heterogeneous disease scenarios, as well as medications histories, an integrative multi-omics approach together with functional validation in gnotobiotic humanized mice help to identify disease-relevant microbiome signatures. We identified metabolic mechanisms that underline microbiota changes in CD patients who either failed to respond, or relapsed following HSCT. Metabolic alterations include the dysregulation of multiple metabolic pathways involved in sulfur dissimilation, assimilation, and bile acid detoxification. The selection of bacterial taxa associated with these metabolic alterations improved the diagnostic classification of CD patients, supporting the idea to implement microbiome signatures for the prediction of disease progression or risk of relapse. In addition, and based on integrated microbiota-metabolite networks, the identification of unknown metabolites could be an approach for mining potential therapeutic targets.

## Methods

### Ethics statement

Mouse experiments and the treatment protocols were approved by the Committee on Animal Health and Care of the local government body of the state of Upper Bavaria (Regierung von Oberbayern; approval number 55.2-1-54-2532-133-2014) and performed in compliance with the EEC recommendations for the care and use of Lab. Animals. (European Communities Council Directive of 24 November 1986 (86/609/EEC). All animals were housed in the germ-free (GF) mouse facility at the Technical University of Munich (School of Life Sciences Weihenstephan).

Hematopoietic stem cell transplantation (HSCT) for treatment of CD refractory to all currently available treatment options is a therapeutic alternative that has been formally approved by the Catalan Office for Transplants (OCATT). All Crohn’s disease patients included in this study were recruited at the Department of Gastroenterology, Hospital Clinic Barcelona. All study methods were approved by the ethics committee of the hospital and complied with all relevant ethical regulations for studies with human research participants. Written informed consent was obtained from all subjects.

### Patient cohort and study design

This paper represents a combined analysis of CD patients recruited within the ASTIC clinical trial (NCT00297193) and the observational trial TrIM, (SAF2012-33560). Autologous Stem Cell Transplantation—International Crohn’s Disease Trial (ASTIC) is a multicenter, prospective, randomized phase III study conducted by the European Crohn’s and Colitis Organization (ECCO), sponsored by the Autoimmune Disease Working Party of the European Group for Blood and Marrow Transplantation (EBMT). The primary and secondary endpoints of ASTIC trial have been published previously^46^. In addition to the ASTIC trial, we recruited patients for expanded microbiome sampling from the “Autologous Hematopoietic Stem Cell Transplant in Crohn’s Disease: genetic, Immune and Microbiome factors involved in disease control (TrIM)” study. This observational trial followed the ASTIC intervention study and was approved by the ethics committee of the institution in Barcelona. TrIM was launched in 2012 to achieve the following main objectives: (a) determining the efficacy of HSCT in patients with refractory CD, improve the safety of the procedure, (b) determining predictive factors of response/remission following HSCT, (c) providing a better understanding of the immune mechanisms underlying achievement of sustained remission following HSCT and (d) sampling stool for compositional (16S rRNA and metagenomic sequencing), functional (fecal transfer of glycerol-preserved samples into germ-free mice) as well as metabolomic analyses. The same study design and transplantation protocol were applied in patients recruited to the ASTIC and TrIM trials. We included detailed information to which cohort each patient belongs to (Supplementary Table S1).

The procedure for performing autologous HSCT in patients with refractory CD was previously described^24,25,47^. Briefly, hematopoietic stem cells are mobilized to peripheral blood by treatment with cyclophosphamide and granulocyte-colony stimulating factor (G-CSF). Peripheral blood cells are then harvested by leukapheresis and frozen. Four weeks later after a lymphoablative regimen of cyclophosphamide and rabbit anti-thymocyte globulin (ATG), cells are thawed and re-infused. The transplantation procedure involves the following phases:

Mobilization phase: all patients undergo peripheral blood stem cell (PBSC) mobilization using the following regimen: 1-h infusion of cyclophosphamide 4 g/m^2^ (2 g/m^2^ on 2 consecutive days) and Filgastrim (non-glycosed G-CSF) 10 μg/kg/day subcutaneously. Administration of filgastrim will commence 5 days after the last cyclophosphamide infusion and end the day before the leukapheresis. Antibiotic regimen during mobilization: levofloxacin prophylaxis (500 mg/day) and fluconazole (400 mg/day) from admission until neutrophil recovery is be administered. When neutrophils are <1.5 × 109/L, doripenem is given at doses of 500 mg every 6 h (in continuous perfusion) until neutrophil recovery. Patients are started on total parenteral nutrition when neutrophils are <1.5 × 109 until neutrophil recovery.

Leukapheresis: leukapheresis is performed on a continuous flow cell separator machine to a target of 3–8 × 106 CD34+ cells/kg body weight.

Conditioning phase: the conditioning regimen consists of intravenous cyclophosphamide 50 mg/kg/day for 4 consecutive days (total 200 mg/kg) and intravenous rabbit anti-thymocyte globulin (rbATG) 2.5 mg/kg/day (total dose 7.5 mg/kg) beginning 2 days after the first dose of cyclophosphamide, for 3 days. To improve tolerability, 500 mg of prednisone are administered before each rbATG infusion. Transplantation is performed 6 days after the start of cyclophosphamide administration and 24 h after the end of rbATG. Antibiotic regimen during conditioning is as follows: levofloxacin prophylaxis (500 mg/day) and fluconazole (400 mg/day) administered from admission until neutrophil recovery. When neutrophils are <1.5 × 109/L, doripenem is given at doses of 500 mg every 6 h (in continuous perfusion) until neutrophil recovery. Patients start on total parenteral nutrition when neutrophils are <1.5 × 109 until neutrophil recovery.

Inclusion criteria include, as previously described^24^, a confirmed diagnosis of active CD at the time of inclusion, active CD defined as a Crohn’s disease activity index (CDAI) >250, objective evidence of active disease based on endoscopic and/or MRI evaluation, and unsatisfactory response to two conventional immunosuppressive agents and two approved anti-TNF antibodies. Patients included in the HSCT must present disease characteristics that are not amenable to surgical treatment due to disease location and or extent and must sign an informed consent. Patients with severe comorbidities, symptoms unrelated to CD inflammatory activity (i.e. stenosis, short-bowel syndrome-related diarrhea), poor compliance or pregnancy were excluded.

Currently, the Inflammatory Bowel Disease Unit, at Hospital Clínic de Barcelona, is the world’s most experienced group in treating CD patients with HSCT. To date 37 patients with refractory CD were evaluated as candidates to receive HSCT. Of those 29 have completed the transplantation, 2 patients are being mobilized, and 7 patients have been excluded for diverse reasons. All patients but one had active disease at the time of transplant. HSCT was followed by achievement of remission without any concomitant therapy in 76% of patients at week 26 post-transplant. Over a follow-up period up to 5 years, treatment-free remission was sustained in ~60% patients at 2 years (*n* = 21) and 50% of patients at 5 years (*n* = 4). Most of the patients who did not improve after HSCT, responded to drugs that had failed before transplant^24^. Antibacterial and antifungal treatment was the same after HSCT for all patients and did not change between remitters and non-remitters. This was assessed in depth in our prior publication^30^. All additional therapies are reported in the ASTIC paper guidelines^48^, as well as in our clinical publications^24,47^.

### Fecal samples from Crohn’s disease patients

From this cohort, fecal samples from 29 patients with up to 5-year follow-up were included. Fresh fecal samples were collected either at the clinic or at home by the patients using a stool collection kit within 24 h prior to the study visit. Patients were instructed to keep the samples stored in the home freezer until transported to the study site. During transport, samples were kept on ice in a cooling bag. Upon arrival to the study site, samples were immediately homogenized in sterile glycerol in PBS (20%) and transferred to the biobank and stored at −80 °C. Cryopreservation in glycerol was ensured to maintain bacterial viability for the transplantation of fecal material in germ-free mice. Clinical assessments with measurement of the Crohn’s disease activity index and biomarkers including C reactive protein and fecal calprotectin were performed at baseline (before HSCT). After discharge, patients were closely followed-up. Crohn’s disease activity index (CDAI) and laboratory markers including CRP, albumin, hemoglobin, ESR, and leukocyte counts were assessed weekly during the first 30 days, and every 6 weeks thereafter. Colonoscopy and/or magnetic resonance were performed at baseline and at weeks 26 and 52 after transplant. Simple endoscopic score for Crohn’s disease (SES-CD) index was used at baseline and during follow-up to assess endoscopic activity. Mucosal healing was defined as SES-CD < 7. MRI index of activity was used at baseline and during follow-up in those patients in whom lesions could not be assessed by ileocolonoscopy; mucosal healing was defined as segmental index <7 in all ileocolonic segments. These data are published^24,30^.

### Selection of donor samples for transfer in germ-free mice

In this cohort of CD patients, fecal samples were collected at three main time points (baseline, week 26 (T26) and week 52 (T52) post-transplant. In addition, fecal samples were collected at various additional time points over time depending on availability and clinical rational. Thus, sample number and time point of sampling per patient varied making a longitudinal analysis difficult. Since the individual disease course also varied in this highly fragile cohort of patients, we generally performed a cross-sectional analysis based on clinical endpoints post-HSCT.

We used information on microbiota composition and stability in addition to disease activity/course and clinical response to HSCT of each individual patient to select representative human donor samples for functional validation in humanized mice. The different disease scenarios included clinical response and non-response to HSCT as well as relapse after HSCT-induced sustained remission. At the level of microbiota analysis, we used cluster stability and community dissimilarity as criteria for sample selection. At this end, we aimed at addressing two main questions:

a. Microbial changes associated with response or failure post-HSCT. Here we focused on two patients where we have the maximal number of longitudinal samples collected overtime, a responder (P#28) and non-responder (P#27).

b. Microbial changes associated with relapse after HSCT-induced long-term remission. Here we focused on one patient where we have full 5-year sampling (P#16).

### Colonization of germ-free mice with human fecal microbiota

Fecal samples processing and preparation were performed under anaerobic conditions. Frozen fecal samples were transferred to a UV-sterilized biosafety hood and pulverized using sterile mortar and pestle while submerged in liquid nitrogen,^21^ to avoid multiple freeze/thaw cycles and to ensure bacterial viability. Preparation of fecal material for inoculation in germ-free mice was done under anaerobic conditions and using reduced PBS (PBS supplemented with 0.05% l-cysteine-HCl) in an anaerobic Coy chamber (atmosphere, 75% N2, 20% CO2, and 5% H2) and vortexed at room temperature for 5 min. The fecal suspension was allowed to settle by gravity for 5 min to exclude residual particulate matter, afterwards, the clear supernatant was transferred under anaerobic conditions into an anaerobic crimped tube, which was transferred to the gnotobiotic facility.

### Animal experiments study design and housing conditions

Germ-free wild-type (WT) and IL-10-deficient (*Il-10*^*−/−*^*)* mice on 129Sv/Ev background were kept at the gnotobiology core facility of the Institute for food and health, Technical University Munich, Germany. Germ-free mice were housed in flexible film isolators ventilated via HEPA-filtered air at 22 ± 1 °C with a 12-h light/dark cycle. Before experiments, littermates were combined and randomly assigned to treatment groups. A maximum of five mice are housed per cage (floor area ~540 cm^2^). Mice received a standard diet (autoclaved V1124-300; Ssniff, Soest, Germany) and autoclaved water ad libitum.

For fecal microbiota transplantation, GF wild-type (WT) and IL-10-deficient (*Il-10*^*−/−*^*/ SvEv129)* male/female mice (8 weeks of age) received 100 µL each of the human fecal suspension via oral gavage (one time, or three times on three consecutive days) using 20 Gauge gavage needle (Fine Science Tools). Human microbiota transplantation experiments were performed in 64 germ-free *IL10*^*−/−*^ and 65 wild-type matching controls. A group of 4–6 Il*-10*^*−/−*^ and 4–6 WT mice were tested per human donor colonization experiment (1x or 3x gavage). Colonized mice were housed in group-specific isolators reserved to mice colonized with the same human microbiota. Mice were killed 4 weeks after colonization.

### Histological scoring

Cecal Swiss-roll tissues were fixed in 4% formaldehyde/PBS for 24 h at room temperature, subsequently dehydrated (Leica TP1020), and embedded in paraffin (McCormick; Leica EG1150C). In total, 5-µm-thick tissue sections were prepared, deparaffinized, and Hematoxylin and Eosin (H&E) staining was performed by using a Leica ST5020 Multistainer system. Scoring of H&E stained tissue sections was performed blindly by single observer through evaluation of lamina propria mononuclear cell infiltration, crypt hyperplasia, goblet cell depletion and architectural distortion as described previously^49^, resulting in a score ranging from 0 (non-inflamed) to 12 (highly-inflamed). Images were acquired by using the Digital microscope M8 and MicroPoint software (PreciPoint GmbH).

### Isolation of immune cells and flow cytometry analysis

MLNs were harvested from humanized mice and dispersed into single-cell suspensions by homogenizing them through a 70-μm nylon cell strainer (BD Biosciences). Cells were washed and resuspended in ice-cold PBS supplemented with FCS (2%, Merck) and EDTA (1 mM, Sigma-Aldrich). Cells were stained and analyzed by using a LSRII system (BD Biosciences). FcR block was done by applying the FcR blocking reagent from Miltenyi following the manufacturer’s instructions. Dead cells were excluded by applying the Zombie GreenTM Fixable Viability Kit (BioLegend). Intracellular staining was performed by using the eBioscience™ Foxp3 / Transcription Factor Staining Buffer Set (Thermo Fisher Scientific). Allophycocyanin-Cy7-conjugated anti-CD3 (17A2, dilution 1:50), PE-Cy7-conjugated anti-CD4 (RM4-5), PE-conjugated anti-CD8 (53-6.7 dilution 1:100), PerCP/Cyanine5.5-conjugated anti-CD62L (MEL-14, dilution 1:100), and Allophycocyanin-conjugated anti-CD25 (PC61.5, dilution 1:100) were from BioLegend. Allophycocyanin-conjugated anti-CD44 (IM7, dilution 1:100) was from BD Pharmingen Biosciences. Data output was subsequently analyzed by using FlowJo software.

### Metagenomic DNA extraction from fecal samples

DNA was extracted from frozen mouse colon content or pulverized human fecal samples by bead-beating followed by a modified version of the protocol by Godon et al.^50^. Briefly, a volume of 600 µL DNA stabilizing solution (Stratec Biomedical, Germany) was added to the fecal aliquots in 2-ml screw-cap polypropylene microcentrifuge tube containing sterile 500 mg Silica beads (0.1-mm-diameter; BioSpec Products) and kept on ice. After the addition of 250 µL of 4 M guanidine thiocyanate – 0.1 M Tris (pH 7.5) and 500 µL of 5% *N*-lauroyl sarcosine – 0.1 M phosphate buffer (pH 8.0), fecal suspensions were vortexed briefly and incubated at 70 °C for 1 h with constant shaking. The mixture was mechanically disrupted by bead beating using a FastPrep®-24 bead beater (MP Biomedicals) supplied with a 24 × 2 mL cooling adapter three times each for 40 s at a speed of 6.5 m/s. An amount of 15 mg of Polyvinylpolypyrroli-done (PVPP, Sigma Aldrich) was added as polyphenol adsorbent and the suspension was centrifuged for 3 min at 15,000×*g* at 4 °C. The supernatant was recovered in a new 2-mL tube and further centrifuged for 3 min at 15,000×*g* at 4 °C. To remove bacterial RNA, a volume of 2 µl RNAse (10 mg/ml) was added to 500 µl clear supernatant and incubated at 37 °C for 30 min with constant shaking. Finally, the genomic DNA was purified using the NucleoSpin® gDNA clean-up kit (Macherey Nagel) following the manufacturer’s instructions. Concentration and purity of the extracted DNA was determined using the NanoDrop® Spectrophotometer ND-1000 (ThermoFisher Scientific, USA). DNA was either used immediately for amplicon analysis or kept frozen as aliquots of 35 µl for metagenomic analysis. Following DNA extraction, all pipetting steps were conducted using a robotized liquid handler to maximize data reproducibility.

### High throughput 16S rRNA gene amplicon sequencing and analysis

After genomic DNA extraction, PCR were conducted in duplicates. DNA was diluted in PCR-grade water (12 ng) and used as template for amplifying (25 cycles) the V3-V4 regions of 16S rRNA genes using primers 341F534 ovh and 785r-ovh (33) in a two-step process shown to minimize bias^51,52^. PCR-fragment concentration was determined using fluorometry and was adjusted to a concentration of 2 nM prior to pooling. Purification of Amplicons was performed using the AMPure XP system (Beckman-Coulter, MA, USA) and sequencing was carried out with pooled samples in paired-end modus (2×250 bpbp) using a MiSeq system (Illumina, CA, USA) according to the manufacturer’s instructions and 25% (v/v) PhiX standard library. To ensure reproducibility among sequencing runs, two samples as negative controls (PCR control without DNA template and a DNA extraction control of 600 µl DNA stabilizer) as well as a positive control (a mock community (ZymoBIOMICS, No. D6300)) were included. 16S rRNA gene sequencing data was preprocessed using the IMNGS pipeline^53^ based on the UPARSE approach^54^. Sequences were de-multiplexed, trimmed to the first base with a quality score <3 and then paired. Sequences with <300 and >600 nucleotides and paired reads with an expected error >3 were excluded from the analysis. Remaining reads were trimmed by five nucleotides on each end to avoid GC bias and nonrandom base composition. The presence of chimeras was additionally tested using UCHIME^55^. Clustering of Operational taxonomic units (OTUs) was done at 97% sequence similarity. OTUs with a relative abundance <0.25% across all samples were removed to exclude spurious OTUs. Taxonomic binning was assigned at 80% confidence level using the RDP classifier^56^ and compared to that of the SILVA ribosomal RNA gene database project^57^. EzBioCloud database was used for precise identification of OTU sequences of interest^58^. Downstream analysis was performed using R-package Rhea^59^. Rarefaction curves were used to estimate sequencing depth. OTUs were normalized and percentage relative abundance was computed. Beta-diversity analysis was used to assess the diversity between groups based on generalized UniFrac distances. Alpha diversity within species was calculated based on species richness and Shannon effective number of species. The contribution of covariates towards differences in the microbial profile of all samples was computed using multivariate permutational analysis using the R function *adonis* from the *vegan package 570v.2.5-6*. The explained variation of a variable is shown in R2 values and are considered significant with a *p*-value ≤ 0.05. Multivariate analysis of metadata (BMI, sex, and family history) co-varying with the available fecal microbiota profiles at baseline showed no statistically significant association. Performing the same analysis on microbial profiles of fecal samples collected longitudinally from all patients showed disease state (R2 = 0.0181924, *p* = 0.003), clinical outcome post-HSCT (R2 = 0.0296583, *p* = 0.001) to be significant. Sex and BMI did not show statistical significance. Between-sample diversity is calculated by generalized UniFrac using GUniFrac v1.1. distances. De-novo clustering is based on Ward hierarchical clustering, the selected number of clusters is based on the Calinski and Harabasz index, performed with the R package NbClust v.3.0. For the analysis of prevalence of categorical variables between groups, a non-parametric Fisher test is used. Correction for multiple testing was performed using the Benjamini–Hochberg false discovery rate control procedure. These statistical analyses have been implemented in pipeline Rhea analysis pipeline^59^. To predict the metagenome functional content from the 16S rRNA gene analysis, we used the Phylogenetic Investigation of Communities by Reconstruction of Unobserved States tool (PICRUSt2)^60^. The PICRUSt results were then analyzed using linear discriminant analysis effect size (LEfSe) to identify microbial functions that were significantly different in their abundance between groups. LEfSe was used to generate the graphs.

### Machine learning methods

The 10-fold cross validated models were performed on two subsets of samples: (a) CD patients with active disease (including patient samples at baseline (*n* = 15), during active disease in relapse or no-remission (*n* = 35)) and patients with inactive disease (patients in remission (*n* = 83)), (b) The same subset but excluding 15 samples collected at baseline.

For each dataset, we ran random forest (RF) models to classify disease state and treatment response separately. OTU tables from each dataset was preprocessed and normalized as described in the 16S rRNA gene sequencing analysis section. Random Forest implemented in the WEKA software suite^61^ was used as a base-classifier and the number of trees was set to 100. The model was evaluated in a 10-fold cross-validation.

### Shotgun metagenomics library preparation

DNA concentrations were measured using Quant-It™ PicoGreen® dsDNA Assay Kit (ThermoFisher Scientific, MA, USA) and a spectrofluorometer (SpectraMax Gemini EM microplate reader Molecular Devices, LLC, USA). DNA purity check was assessed spectrophotometrically (Nano Drop 1000, ThermoFisher Scientific, USA). In total, 200 ng of DNA per sample was sheared using an E220 Focused-ultrasonicator (Covaris® Inc., MA, USA) targeting 300–400 bp fragments following Covaris’s instructions. Metagenomic libraries were constructed using NEBNext® Ultra II™ DNA Library Prep Kit for Illumina®. Dual indexing was done using the kit NEBNext® Multiplex Oligos for Illumina® (Dual index primers set 1, New England BioLabs, UK). Purification and size selection were performed based on Agencourt® AMPure® XP (Beckman-Coulter, MA, USA). Libraries inserts ranged between 400 and 500 bp were evaluated using a Fragment Analyzer™ (Advanced Analytical, IA, USA) using the DNF-474 High Sensitivity NGS Kit (Agilent, Waldbronn, Germany). One sample with sterile water was used as a control for the metagenomics library preparation and sequencing. Libraries quantification were performed using Quant-It™ PicoGreen® dsDNA Assay Kit. Libraries were diluted to 12 pM and 1% PhiX control DNA (Illumina, CA, USA) was spiked in and the libraries were sequenced on an Illumina HiSeq 2500 (Illumina, CA, USA) using the Rapid run paired-end mode (2 × 250 bp).

Quality control: raw metagenome samples (with an average of ~19 million read-pairs per sample) were processed using Trimmomatic version 0.36^62^. First the adapter sequences were removed, retained reads with target length of at least 90 bp and strictness parameter 0.4 (MAXINFO:30:0.4) were further processed to obtain good quality of ~15 million read pairs in average per sample. Reads coding for ribosomal genes were removed from the samples using sortmeRNA version 2.1b^63^ by mapping the quality-controlled samples against SILVA database^64^ version 132 to obtain non-rRNA good quality read-pairs of ~15 million read-pairs per sample in average. Reads related to the human (~12 million read-pairs per sample in average) and mouse genome (~11 million read-pairs per sample in average) were further removed from the samples by mapping against their genomes using hisat2^65^. Finally, PhiX contaminant reads were also removed human (~11 million read pairs per sample in average).

Taxonomic profiling: preprocessed reads from samples from the same treatments were pooled and assembled using megahit version 1.1.3-0^66^. Assembled contigs were used for taxonomic annotation using CAT database^67^, available on (https://github.com/dutilh/CAT). This tool internally uses prodigal v2.6.3^68^ for gene prediction and DIAMOND v 0.9.14^69^ for the alignment against the non-redundant (nr) protein database. Annotated contigs were used as reference and mapped against corresponding samples from each treatment to the reference contigs using bbmap^70^. The taxonomic profile was used as a measure for relative abundance. LEfSe analysis ^71^ was performed from this relative taxonomic abundance profile using http://huttenhower.sph.harvard.edu/lefse/. Those taxa with a Kruskal-Wallis *p*-value <5% and LDA score with at least ×100 fold change (log10 fold change of 2) were considered as potential taxonomic biomarkers.

Functional profiling: gene-annotation was performed for the assembled contigs from each treatment using prodigal v2.6.3^68^. In order to obtain KEGG annotation and to reconstruct KEGG Genes and thereby the functionally associated gene sets named KEGG Modules, the amino acid sequences were used in the KEGG internal annotation tool named GHOSTKOALA^72^. The fraction of KEGG Modules present in a particular treatment was obtained using R package ‘metQy’^73^. This allowed a characterization of the functional capabilities of the microbial communities based on the complete or incomplete presence of functional units. The annotated gene coding sequences obtained from prodigal were used as reference to map for the post-processed reads from the corresponding samples using bbmap to obtain the relative abundance of the KEGG Modules. The taxonomic distribution of KEGG Modules were identified using https://www.kegg.jp/kegg-bin/check_module_taxonomy.cgi and KEGG Modules that were not completely prokaryotic were removed. The relative abundance of KEGG Modules of samples from different treatments was obtained by LEfSe analysis^71^. KEGG Modules with a Kruskal–Wallis *p*-value <5% and LDA score with at least ×10 fold change (log10 fold change of 1) were considered as potential functional biomarkers (http://www.huttenhower.sph.harvard.edu/lefse/).

### Untargeted metabolomics

Untargeted metabolomics measurement was performed using ultra-high-performance liquid chromatography/time-of-flight mass spectrometry. Samples for metabolomics analysis originated from 20 CD patients recruited in (TrIM). Those included 26 fecal samples collected during periods of active disease (baseline or relapse) and 36 samples collected during periods of inactive state disease (remission). Samples from gnotobiotic mice originated from 32 humanized mice (4–6 mice/colonization group; Supplementary Data 4). For sample preparation mouse colon content (20 mg) was mixed with 1 mL methanol-based Dehydrocholic acid extraction solvent (1.3 µmol/L) as an internal standard in a 2 mL bead beater tube (CKMix 2 mL, Bertin Technologies, Montigny-le-Bretonneux, France) filled with ceramic beads (mixture of 1.4 mm and 2.8 mm ceramic beads i.d.). The samples were homogenized by bead beating using a bead beater (Precellys Evolution, Bertin Technologies) supplied with a Cryolys cooling module (Bertin Technologies, cooled with liquid nitrogen) three times each for 20 s with 15 s breaks in between, at a speed of 7322×*g*. Afterwards, the suspension was centrifuged (10 min, 8000 rpm, 10 °C), using a Centrifuge 5415 R (Eppendorf, Hamburg, Germany). Finally, the 100 µL clear supernatant was mixed with 20 µL internal standard solution (*c* = 7 µmol/L) and injected into the LC-TOF-MS system for untargeted analysis. Preparation of human fecal extracts was achieved in a similar manner. Glycerol-preserved human fecal samples (100 mg) were mixed with 5 mL extraction solvent in a 15-mL bead beater tube (CKMix50 15 mL, Bertin Technologies), filled with ceramic beads (mixture of 2.8 mm and 5.0 mm ceramic beads i.d.). The subsequent steps were performed as described above for the mouse colon content samples.

### Targeted bile acid measurement

Targeted bile acid measurement was performed using liquid chromatography-triple quadrupole mass spectrometry (LC−MS/MS). For quantitation a QTRAP 6500 mass spectrometer (Sciex, Darmstadt, Germany) was used in negative electrospray ionization (ESI) mode in combination with Multiple reaction monitoring (MRM) for detection and quantification of bile acids. For detection of the target ions, an ion spray voltage of −4500 V and the following ion source parameters were used: curtain gas (35 psi), temperature (450 °C), gas 1 (55 psi), gas 2 (65 psi), and entrance potential (−10 V). The MS parameters and LC conditions were optimized using commercially available standards of endogenous bile acids and deuterated bile acids, for the simultaneous and unequivocal quantification of selected 34 analytes. For separation of the analytes, a Nexera X2 UHPLC (Shimadzu Europa GmbH, Duisburg, Germany) was used. The system consists of two LC pump systems 30AD, a DGU-20A5 degasser, a SIL-30AC auto-sampler, a CTO-30A column oven and a CBM-20A controller, and equipped with a 100 × 2.1 mm, 100 Å, 1.7 μm, Kinetex C18 column (Phenomenex, Aschaffenburg, Germany). Chromatography was performed with a constant flow rate of 0.35 mL/min using a mobile phase consisted of water (eluent A) and acetonitrile/methanol (3/1, v/v, eluent B), both containing 10 mM ammonium acetate and 0.1% formic acid. The gradient elution started with 32% B for 1.5 min, increased in 4.5 min to 50% B, in 2 min to 60% B, in 1 min to 62% B, increased in 2 min to 80% B, held for 0.5 min, increased in 0.5 min to 100% B; held 2 min isocratically at 100% B, decreased in 0.5 min to the initial ratio of 32% B, followed by 2 min of re-equilibration. The injection volume for all samples was 1 μL, the column oven temperature was set to 40 °C, and the auto-sampler was kept at 10 °C. Data acquisition and instrumental control were performed with Analyst 1.6.2 software (Sciex, Darmstadt, Germany).

### Liquid chromatography-time of flight-mass spectrometry (LC−TOF-MS)

For untargeted LC-MS analysis, an ExionLC UHPLC system (Sciex, Darmstadt, Germany) consisting of two AD UHPLC pumps, a cooled AD auto sampler, an AC column oven and a controller module was connected to a 6600 TripleTOF instrument (Sciex) equipped with an IonDrive ion source (Sciex) operating in positive and negative electrospray mode. After each fifth sample the instruments calibration was verified and corrected using ESI Positive or ESI Negative Calibration solution (Sciex) and a Calibrant Delivery System (Sciex). UHPLC separation was performed using a reversed phase (RP) as well as a hydrophilic interaction (HILIC) stationary phase. RP chromatography was performed on a 100 × 2.1 mm, 100 Å, 1.7 μm, Kinetex C18 column (Phenomenex, Aschaffenburg, Germany) using water (mobile phase A) and acetonitrile (mobile phase B) with 0.1% formic acid each and the following gradient program: 0 min 5% B, 3 min 5% B, 12 min 50% B, 13 min 100% B, 15.5 min 100% B, 16 min 5% B, and 20 min 5% B. For HILIC separation, an ACQUITY BEH Amide column (100 × 2.1 mm, 130 Å, 1.7 μm, Waters, Eschborn, Germany) was applied as stationary phase and a combination of aqueous ammonium acetate and acetonitrile adjusted to pH 3.5 using acetic acid as mobile phase (solvent A: 5 mM ammonium acetate, pH 3.5; solvent B: 5 mM ammonium acetate/acetonitrile 5/95 v/v, adjusted to pH 3.5 prior addition of the acetonitrile) using the following binary gradient program: 0 min 95% B, 3 min 95% B, 12 min 50% B, 13 min 0% B, 15.5 min 0% B, 16 min 95% B, and 20 min 95% B. The total flow of the chromatography was set for both modes to 0.4 mL/min and separation was performed at 40 °C. The mass spectrometer was operated in the SWATH mode with a series of 19 consecutive experiments per 1.4 s measurement cycle. After starting with a high-resolution scan of the intact precursor ions from 50 to 700 *m*/*z* for 200 ms, fragment ions were generated by means of collision-induced fragmentation subsequently for precursor ions within 24 separate windows ranging from 50 to 700 m/z (window width 27 Da each, 1 Da overlap), the resulting fragment spectra were recorded in the high sensitivity mode (50 ms acquisition per window). Ion spray voltage was set at −4500 V in negative and 5500 V in positive mode and the following source parameters were applied: curtain gas 35 psi, gas 1 55 psi, and gas 2 65 psi at temperature 500 °C. Declustering potential was set to 80 V for all experiments while the collision energy was 10 V for precursor ion scans and 35 V including 20 V collision energy spread for the fragmentation in the individual SWATH windows.

### Data analysis and databases used for annotations

Preprocessing: raw data files from UHPLC-TOF-MS were converted into Reifycs Abf (Analysis Base File) files and subsequent untargeted peak picking was performed by means of MS-DIAL software version 3.52^74^ using the SWATH windows reported above. Alignment was performed across the human and mice samples and areas of individual features were exported for further data analysis using the R statistical computing environment. Normalization of peak areas was performed based on quality check samples and the method Q previously reported^75^ for each individual acquisition method (positive and negative electrospray ionization, reversed phase (RP), and hydrophilic interaction (HILIC) separation methods). Quality check (QC) samples were used to correct for intensity changes along the injection sequence using method Q. QC samples were injected after every fifth sample and were prepared by mixing aliquots (20 µL each) of every sample. Median relative standard deviations (RSD) were subsequently determined for every feature in the QC samples and median values of RSDs were reported for each method. (RSD) for RP (positive) and (negative) measurement modes was 8.6 and 3.5, respectively. While for HILIC (positive) and (negative) modes, it was 7.4 and 5.2, respectively. All features were combined into a single feature table. Metabolite data obtained by LC-MS were adjusted after peak picking by its Total Ion Current (TIC). For each feature in a sample the intensity value is divided by total intensity for the sample. Given a constant biomass in fecal samples (100 mg for human samples and 20 mg for mouse samples), a similar summed TIC can be expected. Normalization of TIC between samples therefore can partly adjust for the different water content in fecal samples, as a lower TIC is assumed here to be indicative for a higher water content. Near-zero variance MS features were subsequently removed using the caret package (R package version 6.0-71, www.CRAN.R-project.org).

### Multivariate modeling of metabolomics data

Unsupervised (PCA) and supervised multivariate data analysis of metabolomics data (PLS-DA) was performed by application of the ropls package^76^, while integrative analysis of microbiome and metabolome data was based on the methodologies used in the boruta and mixomics package within R^77,78^. The data-independent MS/MS SWATH mode was applied due to its capability to acquire all MS2 spectra and the high quantitative accuracy. Preprocessing was performed by filtering out near-zero-variance features, resulting only in a negligible reduction of the total feature number. Furthermore, feature selection for integration of metagenomics and metabolomics information was not based on the PLS-DA model, instead fold change and significance as calculated for the volcano plots were used as selection criteria. The total count of metabolomics features in the multi-omics models was therefore 1252 (mice) and 451 (human). The predictive performance of the PLS-DA model is assessed by the cumulative Q2Y metric which can have values between 0 and 1. The higher the Q2Y, the better the performance. The Q2Y value for the human dataset was (0.64) and for the mouse dataset (0.839). To estimate the significance of Q2Y (and R2Y), permutation testing^79^ was used. Additional models were built after random permutation (*n* = 1000) of the outcome variable (disease activity), and Q2Yperm are computed and compared to the Q2Y value. The *p*-value is equal to the proportion of Q2Yperm above Q2Y value. Cross-validation of the PLS-DA models was performed using the RVAideMemoire package within the R programming environment. Metabolite features selected after volcano plot visualization based on *p*-value and fold-change, as well as OTU count data pre-filtered to remove features with a low sum of counts 0.01% (adapted from Arumugam et al.^80^) followed by total sum scaling and Centered Log Ratio transformation (CLR) were used for integrative multi-omics analysis. Mapping of metabolite features containing a potential sulfate moiety was based on filtering for tandem mass spectra containing a fragment with *m*/*z* 97 representing the [HSO4]-ion with at least 10% intensity and a raw spectral count of 100. As additional confirmation step, spectra were checked for presence of a fragment with 79.96 (±0.01) indicating [SO3]-fragment ions^81^.

### Multi-omics data integration

For the multi-omics data integration, the first step inputs multiple omics datasets measured on the same individual, that were previously normalized and filtered. This is followed by a multivariate dimension reduction method that seeks for latent components – linear combinations of variables from each omics dataset, that are maximally correlated as specified in a design matrix. The design matrix indicates which datasets should be connected such that their pairwise correlations are maximized. The identification of a multi-omics panel is performed via penalties that shrink the variable coefficients defining the latent components to zero. The performance of the model and associated multi-omics panel is then assessed using cross-validation repeated several times to ensure reliable evaluation and the balanced error rate (BER) or area under the receiver operating curve (AUC) are reported.

### Statistical analysis

Statistical analysis was performed with GraphPad Prism (version 7.00; GraphPad Software, San Diego, CA). For comparison between two groups, Student’s two-tailed unpaired *t* test was used. For comparison between more than two groups one-way ANOVA followed by pairwise comparison testing (Bonferroni post-hoc test). *P* < 0.05 was considered significant. *p < 0.05; **p < 0.01; ***p < 0.001. Data is presented as mean ± SD.

### Reporting summary

Further information on research design is available in the Nature Research Reporting Summary linked to this article.

## Supplementary information

Supplementary Information

Description of Additional Supplementary Files

Supplementary Data 1

Supplementary Data 2

Supplementary Data 3

Supplementary Data 4

Supplementary Data 5

Supplementary Data 6

Reporting Summary

## Data Availability

The demultiplexed reads for all 16S amplicon sequencing datasets from both patients and gnotobiotic humanized mice have been deposited to the NCBI Sequence Read Archive [http://www.ncbi.nlm.nih.gov/sra] under the accession No. PRJNA565903 and No. PRJNA565980, respectively. Metagenomics sequences generated during this study have been deposited at SRA NCBI under the accession No. PRJNA575186. Full list of identified metabolite features is included as Supplementary Data 5. Raw peak areas of metabolomics data generated during this study have been enclosed as Supplementary Data 6 to the manuscript. Source data are provided with this paper.

## Source data

Source Data

## Code availability

The custom code for shotgun metagenomics data analysis is available at https://github.com/HallerLab/Metagenomics-Metwaly-et-al.-2020. The software packages used for metabolomics analysis are free and open source, including mixOmics methods available via https://github.com/cran/mixOmics. 
