# Supplementary material for: Integrated microbiota and metabolite profiles link Crohn’s disease to sulfur metabolism
Source: Nat Commun. 2020 Aug 28;11:4322. doi: 10.1038/s41467-020-17956-1 (PMC7456324; doi:10.1038/s41467-020-17956-1)
Supplement: Supplementary file 1 — Supplementary Information [file 41467_2020_17956_MOESM1_ESM.docx]

**Integrated microbiota and metabolite profiles link Crohn’s disease to sulfur metabolism**

**Metwaly et al.**

**Supplementary figures and tables**

**
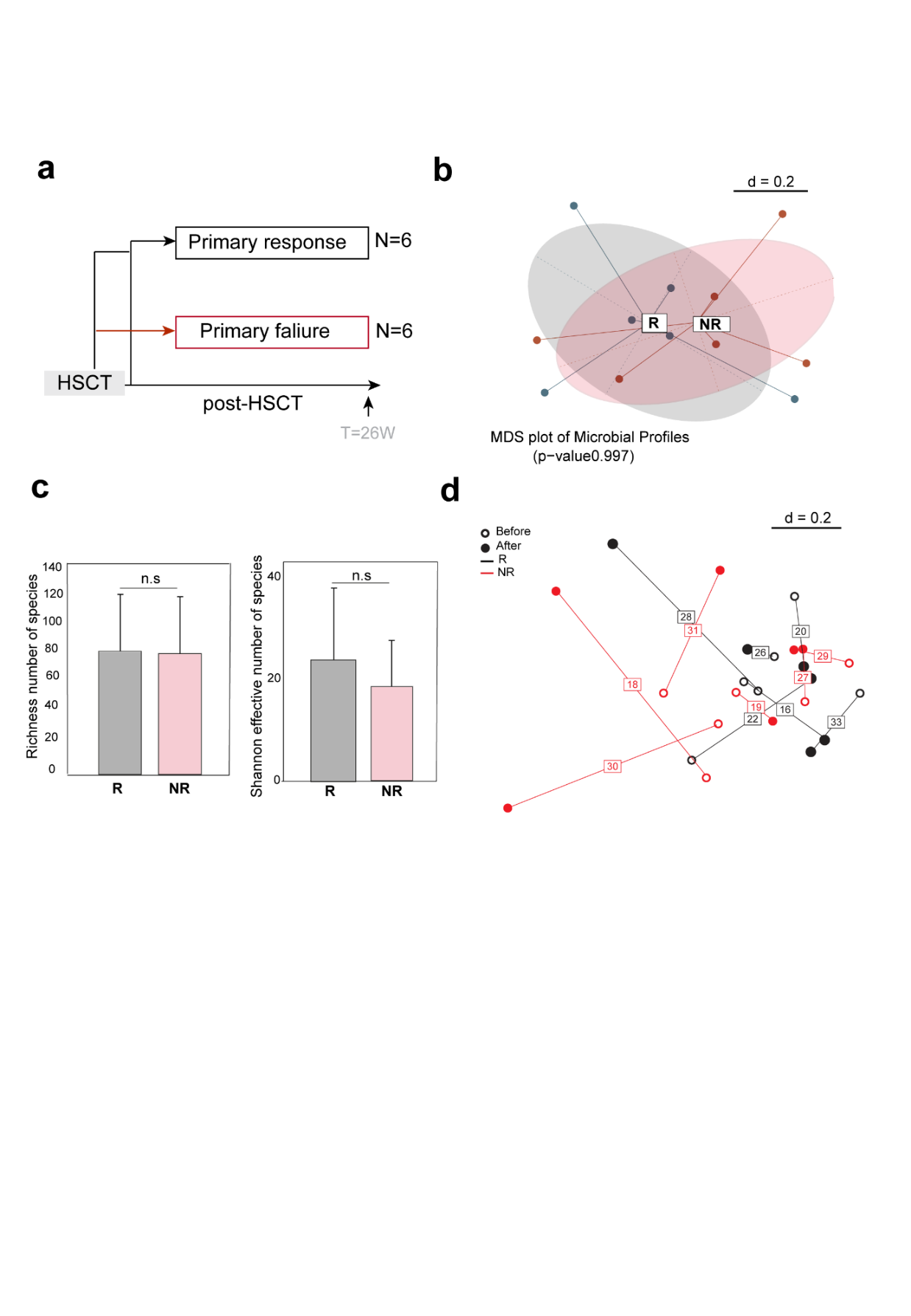
**

**Supplementary Figure 1: Microbial community diversity and richness at baseline could not predict response to HSCT therapy(a)** Responders and non-responders included in the analysis **(b)** MDS plot of bacterial composition of CD patients stool samples collected at baseline from patients who are either responders (n=6) or non-responders (n=6) post-HSCT. Beta-diversity analysis showed no significant difference between responders and non-responders at baseline. Permutational multivariate analysis of variance (PERMANOVA) using distance matrices (vegan:adonis) was performed to determine if the separation of the groups is significant.**(c)** Species richness and diversity in responders and non-responders at baseline. Data plotted as mean values ±SD. Statistical significance was tested using paired sample *t*-test. P-value < 0.05 is considered significant. **(d)** Generalized UniFrac distances in responders and non-responders before and after HSCT.


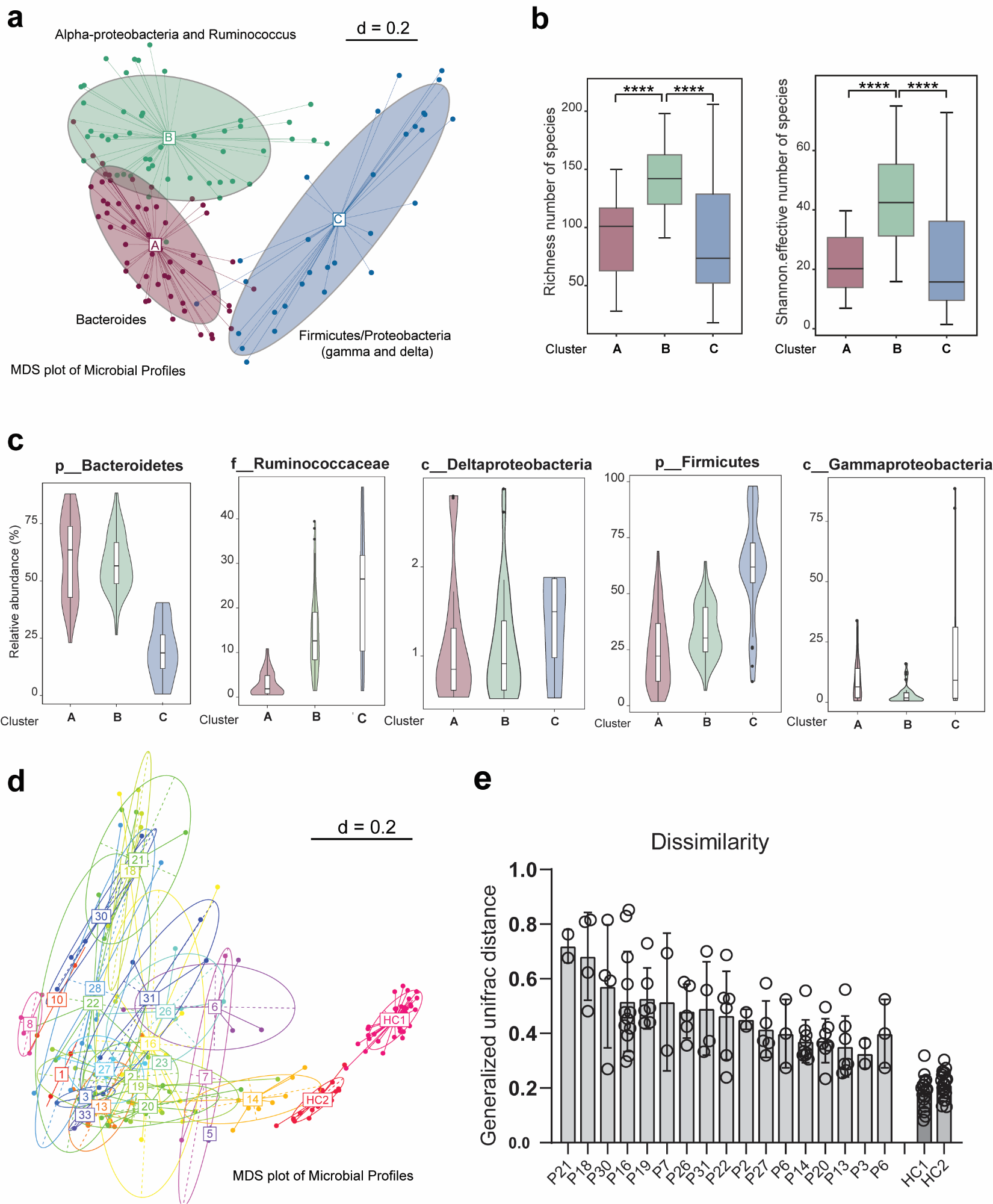


**Supplementary Figure 2: Selection of human donors for functional validation in humanized mice**

**(a)** Unsupervised clustering using *k*-means and Calinski–Harabasz index resulted in 3 clusters (A, B and C). **(b)** Species richness and diversity of each cluster. For boxplots, 5–75 percentiles are shown. The upper whisker extends from the hinge to the largest value no further than 1.5 * IQR from the hinge (where IQR is the inter-quartile range, or distance between the first and third quartiles). The lower whisker extends from the hinge to the smallest value at most 1.5 * IQR of the hinge. Data beyond the end of the whiskers are outliers and are plotted individually. Significance is calculated by Mann-Whitney test. *p ≤ 0.05, **p ≤ 0.01, ***p ≤ 0.001 **(c)** Unsupervised clustering resulted in 3 robust clusters characterized by abundance of signature taxa. **(d)** Visualization of the multidimensional distance matrix in individual CD patients and in the two healthy individuals (HC1 and HC2). Multi-dimensional scaling plot (MDS) based on generalized UniFrac distances, where each circle represents an individual microbial composition and each color represents samples collected from every patient longitudinally over time. A permutational multivariate analysis of variance using distance matrices (vegan:adonis) was performed in each case to determine if the separation of groups is significant, as a whole and in pairs. **(e)** Microbial community dissimilarity shown by generalized UniFrac distances. Distances were computed based on pairwise comparisons within samples collected longitudinally from each CD patient (each number refers to one patient ID). The same analysis was performed on fecal samples collected longitudinally over 20 consecutive days from 2 healthy subjects (HC1 and HC2). Distances are plotted as mean values ±SD. No statistical analysis performed. Most individual patients showed higher overall community dissimilarity compared to the two healthy subjects.

**
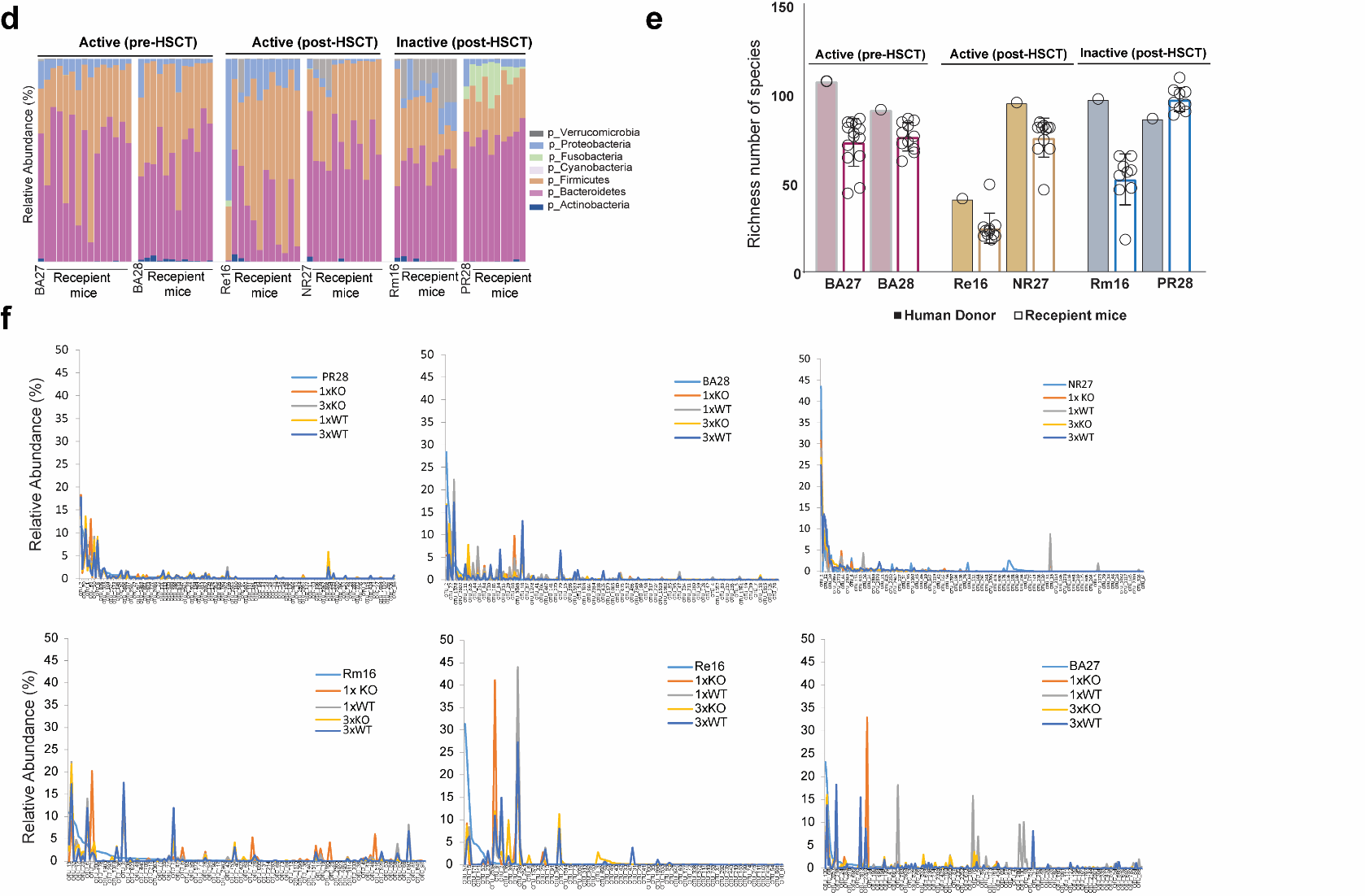

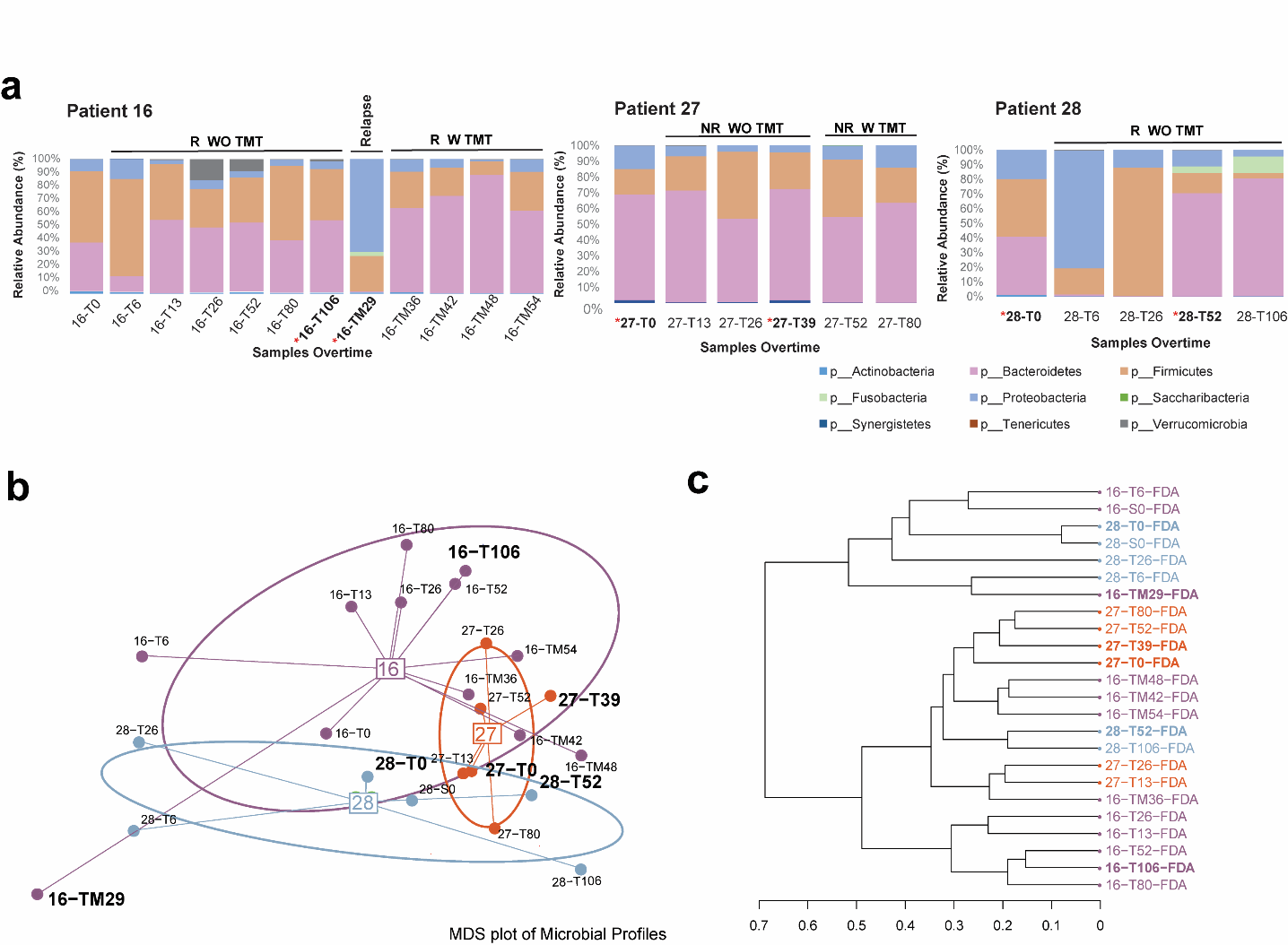
**

**Supplementary Figure 3: Humanized mice reflect the microbial dysbiotic features of their respective human donors**

**(a)** Individual Patient’s clinical course and microbial composition of fecal samples collected before and over-time after HSCT. Two fecal samples per patient were selected for transplantation in GF mice (bold, asterisk). **(b)** MDS of fecal microbiota showing the compositional shifts in samples collected longitudinally in the 3 representative patients selected for the humanization experiments. Two donor samples were used for transplantation in germ-free mice (Bold). **(c)** A corresponding phylogram based on the Ward’s minimum variance method showing the hierarchical clustering of samples. **(d)** Taxonomic composition of human donors and respective humanized mice showing similarities at phyla level. **(e)** Humanized mice reflect the community richness of their original human donors. For each colonization experiment, the analysis is based on fecal microbiome of one human donor (n=1) and the respective humanized mice (BA27, n=14 , BA28, n=11 , Re16, n=12 , NR27, n=11 , Rm16, n=9 and PR28, n=9). Mouse data plotted as mean values ±SD, showing similar trends. No significance analysis performed. **(f)** Histograms showing the selective enrichment of certain human bacterial taxa when transferred into germ-free mice, as shown by the relative abundance of OTUs in original donor microbiota (blue) and respective humanized mice (red) in both genotypes (WT and KO) and using the different humanization protocols (1x or 3x gavage). Data shown as % relative abundance of OTUs in in an inactive or an active state of disease. **Abbr.:** BA, Baseline active; PR, Primary responder; NR, non-responder; Rm, Remission; Re, Relapse


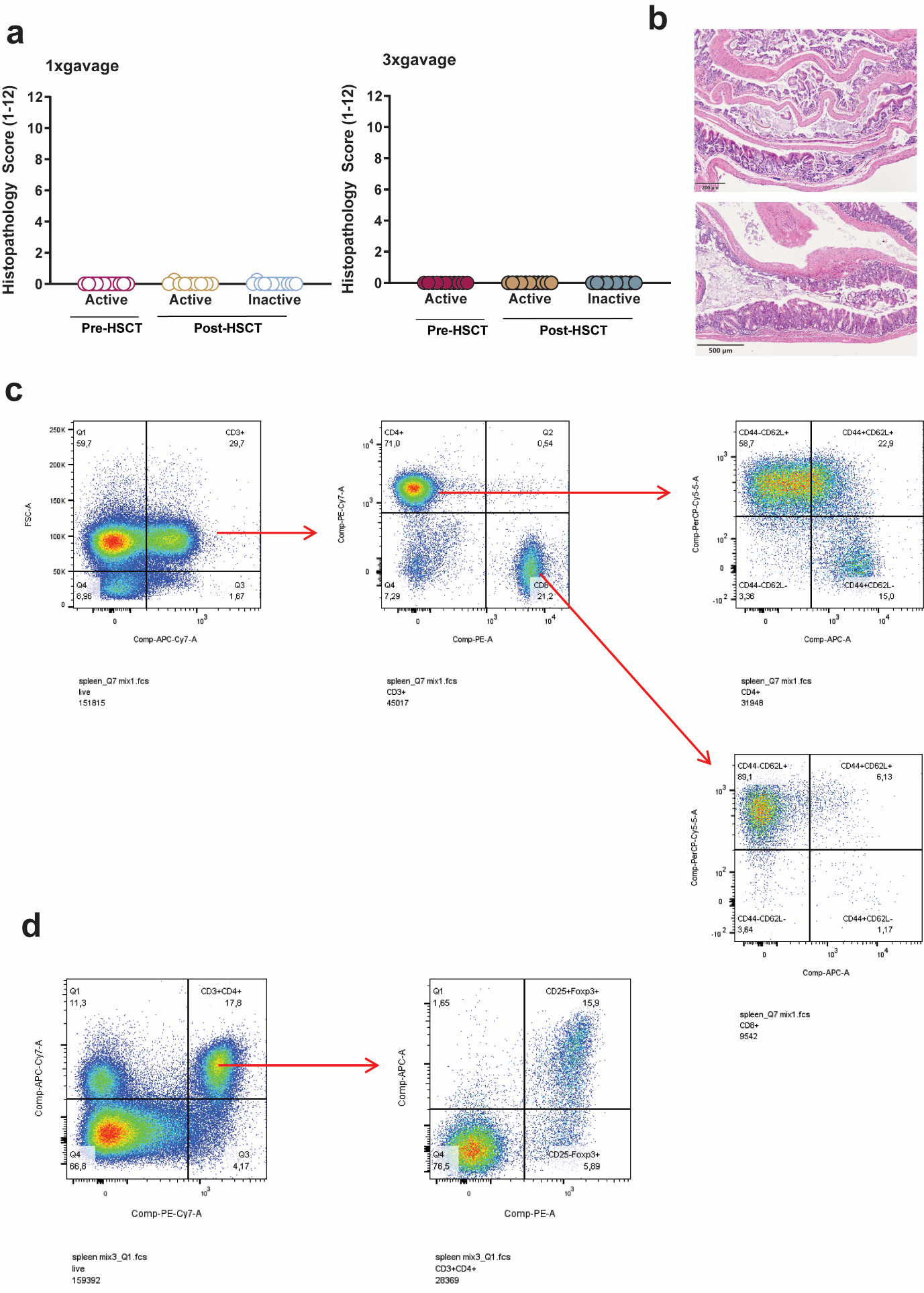


**Inactive (post-HSCT)**

**Active (post-HSCT)**

**Supplementary Figure 4:** **Humanized mice reflect disease states of their respective human donors**

**(a)** Histopathology of cecum tissue in ex-germfree wildtype mice colonized one time (open symbols) with microbiota from CD patients at baseline (n=12), during active disease post-HSCT (n=9) and during inactive disease post-HSCT (n=12). **(d)** Histopathology of cecum tissue in ex-germfree wildtype mice colonized three times (closed symbols) with microbiota from CD patients at baseline (n=11), during active disease post-HSCT (n=11) and during inactive disease post-HSCT (n=10). **(b)** Representative H&E staining of cecum tissue in ex-germfree wildtype mice, colonized with microbiota from patients with active or inactive disease. H&E staining was performed on tissues from all mice included in the analysis. One representative picture is included. **(c)** Gating strategy for flow cytometry analysis. **(a)** representative nested gating strategy illustrating lymphocyte population being subgated to the level of CD62L^high^CD44^low^ (native T cells) CD62L^low^CD44^high^ (effector memory T cells) and central memory T cells (T_CM_), CD62L^high^CD44^high^ (**d)** representative gating strategy illustrating lymphocyte population being sub-gated to CD4^high^CD25^high^Foxp3^high^ CD4^high^CD25^low^Foxp3^high^ regulatory T-cells.


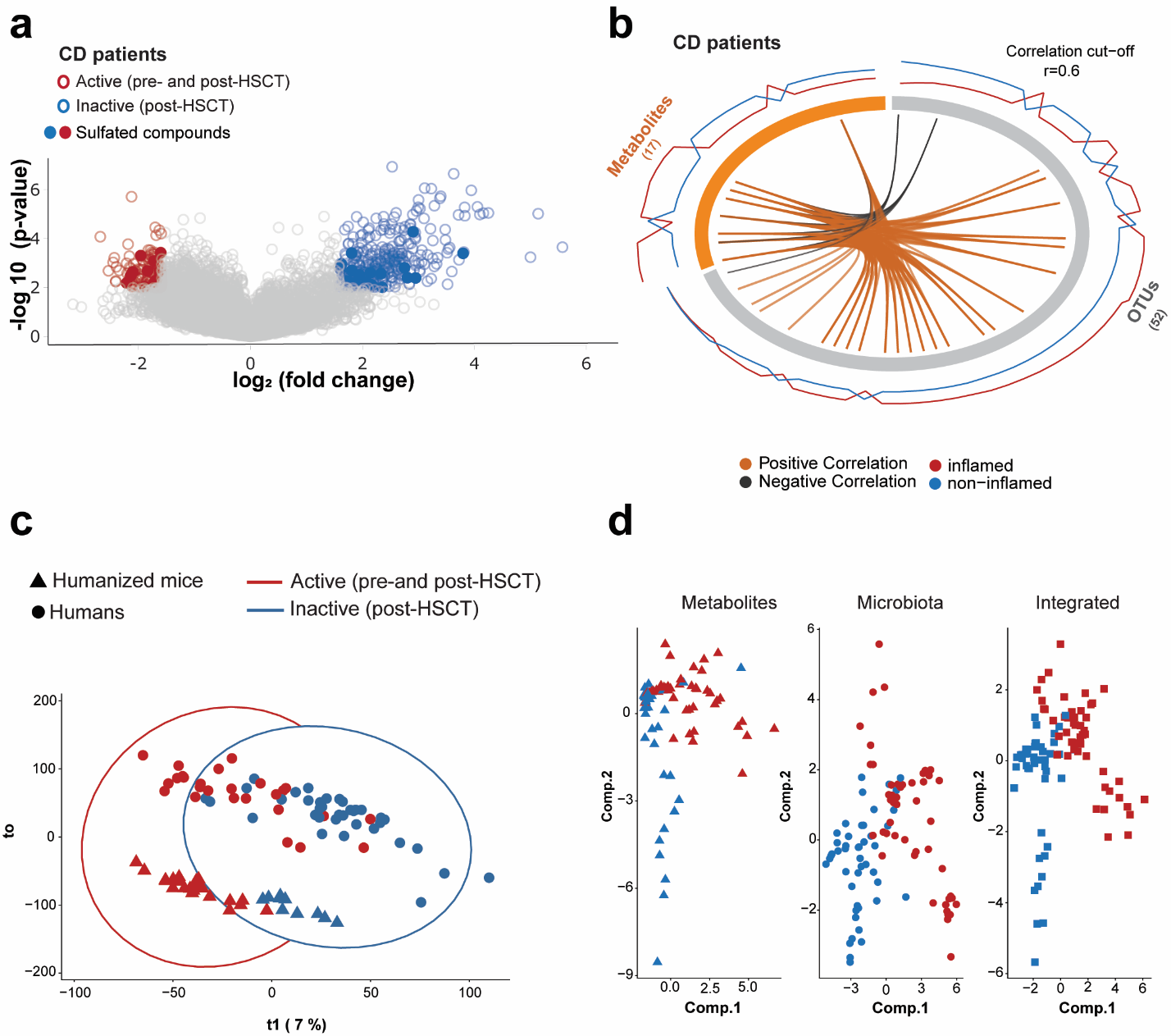


**Supplementary Figure 5: Metabolic profiling of CD patients and humanized mice**

**(a)** Volcano plot of differentially abundant metabolites in CD patients gut microbiome selected based on fold change (log2 >3). Sulfated compounds are highlighted. **(b)** Circos plot on CD patients integrated fecal metabolome-microbiome dataset showing the positive (negative) correlation (r >= 0.6) between selected features as indicated by the orange (black) links **(c)** OPLS-DA score plot showing separation of metabolite profiles between human donors with different disease states, as well as humanized mice colonized with human microbiota. Significance in the OPLS-DA model was tested using cross-validation (CV) analysis of variance using the RVAideMemoire package within the R programming environment **(d)** Separation of CD patients and humanized mice according to disease activity post-HSCT based on metabolite features, microbiota and on the integrated microbiome-metabolome dataset.

**
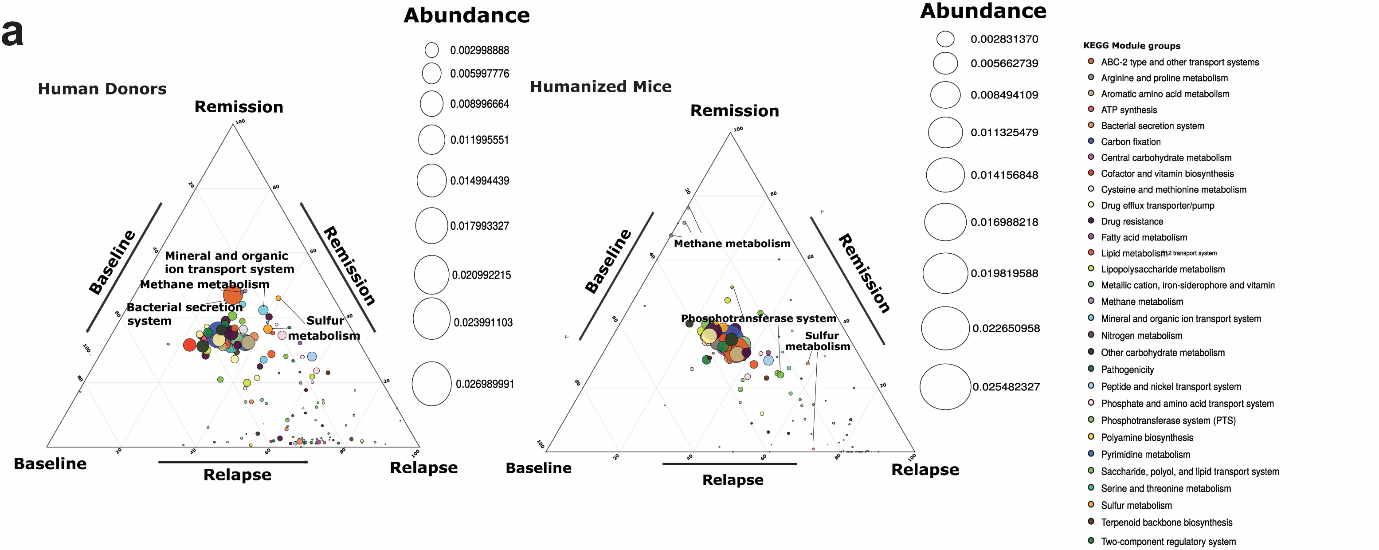
**

**
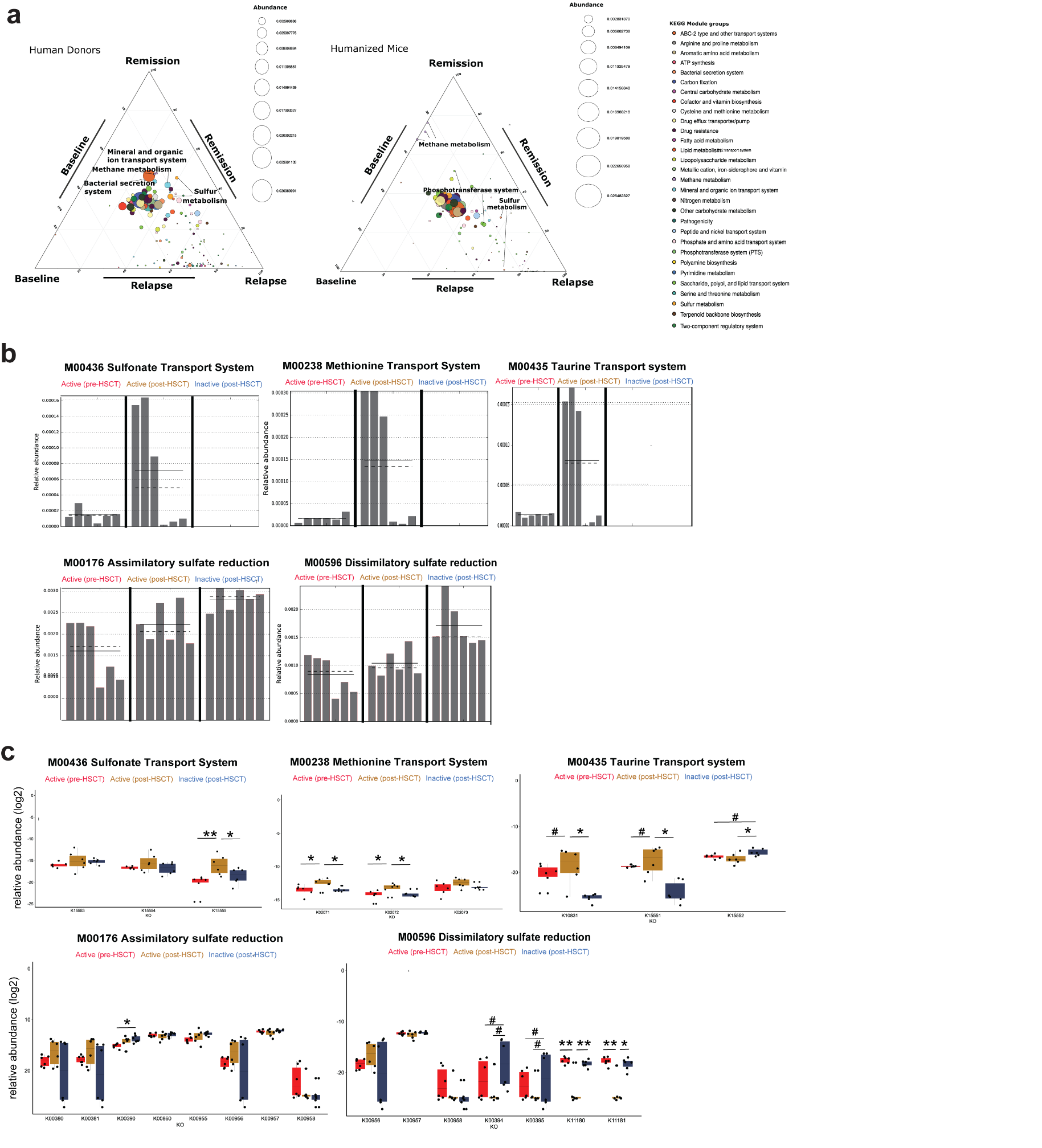
**

**Supplementary Figure 6: Shotgun metagenomics identify sulfur metabolism pathways as key mechanisms linked to disease**

**(a)**Ternary plots: KEGG modules that are significantly different amongst the conditions under study were picked based on LEfSe analysis. Median relative abundance of KEGG modules for Baseline, Remission-responder and Relapse-non-responder conditions in human donors or humanized mice was plotted. The color represents the classes encompassing the KEGG modules (Class_III given by metq package in R for KEGG Modules). The size of the bubble represents the overall average relative abundance of the KEGG Module. **(b)** Relative abundance of differentially abundant KEEG modules in humanized mice colonized with microbiota from CD patients with active disease (pre-HSCT), (post-HSCT) or with inactive disease (post-HSCT). Analysis show high relevance of sulfur associated KEEG modules. Data represented as histograms of the relative abundance of each functional module. The solid line shows the average, and the dotted line shows the median of individual samples representing each of the three conditions. Analysis is based on (n=3) mice per group, with a total of (n=18) mice colonized with human microbiota from 3 different donors (and 6 different microbial communities). The relative abundance (y-axis) is represented as a fraction of 1. **(c)** Relative abundance of sulfur -associated KEGG Modules that are at least 10x fold change with p-value <5% from LEfSe analysis. Statistically significant KEGG ortholog in each KEGG Module (ANOVA p-value) is represented by symbols denoted by ‘***’,’**’, ‘*’,’#’. Statistical significance of the difference in KEGG ortholog abundance amongst the conditions under study (Tukey’s honest significant adjusted p-value) is denoted by the symbols ‘***’,’**’,‘*’,’#’ **#**0.05<p-value<0.1, *p≤ 0.05, **p<0.01, ***p<0.001.

| Patient ID | Response | ASTIC | No. samples | Week 0 | | Week 26 | | Week 52 | |
| --- | --- | --- | --- | --- | --- | --- | --- | --- | --- |
|  |  |  |  | CDAI | SESCD | CDAI | SESCD | CDAIW52 | SESCD |
| 1 | YES | Yes | 2 | 225,00 | 8 | 570,00 | na | 291,00 | 3 |
| 2 | NO | Yes | 3 | 284,00 | 13 | 126,50 | na | 246,00 | 25 |
| 3 | YES | Yes | 3 | 256,00 | 14 | 48,00 | na | 70,00 | 1 |
| 5 | YES | Yes | 2 | 373,00 | 13 | 173,00 | na | 144,70 | 1 |
| 6 | NO | Yes | 4 | 326,00 | 24 | 106,70 | na | 94,40 | 12 |
| 7 | YES | No | 3 | 364,50 | 2 | 53,14 | na | 53,14 | 1 |
| 8 | YES | No | 4 | 347,80 | 7 | 155,17 | na | 287,00 | 3 |
| 9 | NO | No | 1 | 231,00 | 18 | 272,00 | na | 296,00 | 12 |
| 10 | YES | No | 2 | 347,00 | 23 | 52,80 | na | 151,80 | 5 |
| 11 | YES | No | 1 | 269,00 | 15 | 224,00 | na | 117,00 | 1 |
| 12 | NO | Yes | 1 | 419,00 | 36 | 72,76 | na | 174,45 | 24 |
| 13 | YES | Yes | 8 | 332,00 | 26 | 137,00 | na | 88,22 | 2 |
| 14 | YES | No | 10 | 262,10 | 34 | 61,50 | 4 | 24,74 | 0 |
| "15/23" | YES | No | 3/4 | 336,64 | 19 | 101,00 | 6 | 1,50 | 0 |
| 16 | YES | Yes | 12 | 236,00 | 19 | 180,29 | 6 | 54,00 | 0 |
| 18 | NO | No | 5 | 267,70 | 16 | 232,00 | 9 | 264,80 | 16 |
| 19 | NO | No | 7 | 450,00 | 22 | 390,00 | 21 | 382,00 | 21 |
| 20 | YES | No | 8 | 162,89 | na | 94,00 | na | 164,78 | na |
| 21 | NO | No | 3 | 204,98 | 32 | 321,00 | 29 | 77,00 | na |
| 22 | YES | No | 8 | 278,66 | 20 | 70,76 | 4 | 36,00 | 0 |
| 25 | YES | No | 1 | 149,37 | na | 40,90 | na | 153,10 | na |
| 26 | YES | No | 6 | 335,00 | 12 | 14,00 | 0 | 10,00 | 0 |
| 27 | NO | No | 6 | 448,31 | 22 | 290,00 | 11 | 417,00 | 12 |
| 28 | YES | No | 6 | 232,00 | 18 | 153,98 | 4 | 32,84 | 0 |
| "29/35" | NO | No | 3/4 | 324,60 | 21 | 31,90 | 14 | 278,92 | 32 |
| 30 | NO | No | 5 | 133,80 | 10 | 98,40 | 7 | 87,60 | 7 |
| 31 | NO | No | 5 | 123,40 | 34 | 122,88 | 32 | 37,20 | 27 |
| "33/36" | YES | No | 3 | 126,69 | 9 | 28,93 | 0 | 29,76 | 0 |
| 34 | NO | No | 1 | 134,35 | na | 14,90 | na | 10,22 | na |

**Supplementary Table S1:** Metadata as well as clinical and endoscopic disease activity of CD patients at baseline and during follow-up.

ASTIC, “Autologous Stem Cell Transplantation in Refractory Crohn's Disease” clinical trial

CDAI, Crohn’s Disease Activity Index

SES-CD, Simple Endoscopic Score for Crohn’s Disease.

| Patient ID | BMIT0 | BMIW26 | BMIW39 | BMIW52 | BMIW80 | BMIY2 | BMIY3 | BMIY35 | BMIY4 | BMIY45 | BMIY5 |
| --- | --- | --- | --- | --- | --- | --- | --- | --- | --- | --- | --- |
| 1 | 29.47 | 28.10 | 28.00 | 30.00 | 31.00 | 32.00 | 28.9 | 27.16 | 26.79 | 27.68 | 27.68 |
| 2 | 24.70 | 23.00 | 22.80 | 21.00 | 24.00 | 24.00 | 22.4 | 22.14 | 21.04 | 22.29 | 23.00 |
| 3 | 29.20 | 28.70 | 29.00 | 29.38 | 27.50 | 32.00 | 31.9 | 29.58 | 25.75 | 30.53 | 30.53 |
| 5 | 21.00 | 21.00 | 20.94 | 19.24 | 20.56 | 18.90 | 19.0 | 19.00 | 20.00 | na | na |
| 6 | 21.60 | 21.90 | 21.96 | 22.00 | 22.77 | 22.61 | 22.4 | 21.96 | 20.48 | na | na |
| 7 | 19.69 | 22.39 | 22.39 | 21.99 | 22.39 | 22.30 | 21.8 | na | 22.77 | 21.93 | 21.49 |
| 8 | 18.00 | 20.50 | 22.12 | 20.44 | 25.30 | 25.00 | 27.3 | 27.40 | 27.90 | 27.90 | 27.39 |
| 9 | 31.16 | 35.50 | 36.70 | 37.17 | 31.79 | 30.34 | na | na | na | na | na |
| 10 | 20.48 | 21.95 | 21.74 | 21.75 | 21.20 | 19.92 | 21.5 | 21.74 | 21.25 | 21.60 | 21.35 |
| 11 | na | 29.28 | 29.20 | 27.75 | 24.98 | 24.98 | na | na | na | na | na |
| 12 | 18.60 | 23.85 | 23.00 | 23.69 | 22.12 | 22.94 | 24.8 | 24.84 | na | na | na |
| 13 | 28.00 | 29.83 | 32.37 | 32.50 | 32.20 | 31.00 | 32.2 | 34.10 | 33.49 | 34.77 | 35.94 |
| 14 | 24.40 | 21.73 | 23.41 | 23.41 | 27.42 | 29.00 | 30.6 | 31.11 | 31.11 | 29.87 | 30.74 |
| 15 (23) | 19.25 | 22.00 | 24.26 | 23.52 | 22.98 | 23.81 | 24.2 | 23.50 | 23.44 | na | na |
| 16 | 21.87 | 21.87 | 21.99 | 21.00 | 21.10 | 20.90 | 21.5 | 22.30 | 20.47 | 21.25 | na |
| 18 | 16.27 | 17.50 | 18.00 | 17.00 | 20.21 | 20.21 | 20.7 | 20.72 | 19.98 | 20.05 | na |
| 19 | 40.63 | 38.53 | 41.50 | 43.38 | 47.75 | 57.00 | na | na | na | na | na |
| 20 | 22.90 | 23.98 | 24.86 | 25.18 | 18.90 | 20.83 | 22.5 | 24.49 | 24.86 | na | na |
| 21 | 19.87 | 16.98 | 19.00 | 20.06 | na | na | na | na | na | na | na |
| 22 | 15.02 | 22.00 | 24.00 | 25.15 | 24.51 | 23.15 | 22.0 | 22.92 | na | na | na |
| 25 | 16.98 | 17.60 | 18.93 | 18.93 | 17.75 | 17.06 | 17.5 | na | na | na | na |
| 26 | 29.10 | 33.98 | 34.18 | 32.89 | 30.90 | 32.34 | 32.9 | na | na | na | na |
| 27 | 38.79 | 39.13 | 39.13 | 39.93 | 41.87 | 34.36 | na | na | na | na | na |
| 28 | 22.97 | 25.07 | 26.22 | 24.78 | 26.87 | 25.89 | na | na | na | na | na |
| 29 (35) | 21.47 | 19.49 | 20.45 | 19.49 | 20.81 | 21.14 | na | na | na | na | na |
| 30 | 18.52 | 19.68 | 19.41 | 19.41 | 20.02 | 19.68 | na | na | na | na | na |
| 31 | 15.58 | 16.81 | 16.84 | 17.80 | 20.66 | 21.88 | na | na | na | na | na |
| 33 | 17.81 | 19.10 | 20.55 | 20.79 | na | na | na | na | na | na | na |
| 34 | 19.92 | 23.51 | 22.53 | 23.12 | 23.46 | na | na | na | na | na | na |

**Supplementary Table S2:** Body mass index (BMI) data of CD patients at baseline and during follow-up

| Patient_ID | Time (T=weeks, TM=months) | Endoscopic_Activity | Status |
| --- | --- | --- | --- |
| 1 | TM48 | R W TMT | Inactive post_HSCT |
| 1 | TM60 | R W TMT | Inactive post_HSCT |
| 2 | TM46 | R W TMT | Inactive post_HSCT |
| 2 | TM54 | R W TMT | Inactive post_HSCT |
| 2 | TM60 | R W TMT | Inactive post_HSCT |
| 3 | TM48 | R WO TMT | Inactive post_HSCT |
| 3 | TM54 | R WO TMT | Inactive post_HSCT |
| 3 | TM60 | R WO TMT | Inactive post_HSCT |
| 5 | TM36 | R WO TMT | Inactive post_HSCT |
| 5 | TM48 | R WO TMT | Inactive post_HSCT |
| 6 | T106 | NR W TMT | Active post-HSCT |
| 6 | TM30 | NR W TMT | Active post-HSCT |
| 6 | TM36 | NR W TMT | Active post-HSCT |
| 6 | TM42 | NR W TMT | Active post-HSCT |
| 7 | T106 | R WO TMT | Inactive post_HSCT |
| 7 | TM54 | R W TMT | Inactive post_HSCT |
| 7 | TM60 | R W TMT | Inactive post_HSCT |
| 8 | T106 | R W TMT | Inactive post_HSCT |
| 8 | TM36 | R W TMT | Inactive post_HSCT |
| 8 | TM42 | R W TMT | Inactive post_HSCT |
| 8 | TM48 | R W TMT | Inactive post_HSCT |
| 10 | T106 | R WO TMT | Inactive post_HSCT |
| 10 | TM36 | R WO TMT | Inactive post_HSCT |
| 11 | T80 | R WO TMT | Inactive post_HSCT |
| 12 | T80 | NR W TMT | Active post-HSCT |
| 13 | T106 | R WO TMT | Inactive post_HSCT |
| 13 | T52 | R WO TMT | Inactive post_HSCT |
| 13 | TM30 | R WO TMT | Inactive post_HSCT |
| 13 | TM36 | R WO TMT | Inactive post_HSCT |
| 13 | TM42 | R WO TMT | Inactive post_HSCT |
| 13 | TM48 | NR WO TMT | Active post-HSCT |
| 13 | TM54 | NR WO TMT | Active post-HSCT |
| 13 | TM60 | R W TMT | Inactive post_HSCT |
| 14 | T106 | R WO TMT | Inactive post_HSCT |
| 14 | T26 | R WO TMT | Inactive post_HSCT |
| 14 | T52 | R WO TMT | Inactive post_HSCT |
| 14 | T80 | R WO TMT | Inactive post_HSCT |
| 14 | TM30 | R WO TMT | Inactive post_HSCT |
| 14 | TM36 | R WO TMT | Inactive post_HSCT |
| 14 | TM42 | R WO TMT | Inactive post_HSCT |
| 14 | TM48 | R WO TMT | Inactive post_HSCT |
| 14 | TM54 | R WO TMT | Inactive post_HSCT |
| 14 | TM60 | R WO TMT | Inactive post_HSCT |
| 15 | T13 | R WO TMT | Inactive post_HSCT |
| 15 | T26 | R WO TMT | Inactive post_HSCT |
| 15 | T52 | R WO TMT | Inactive post_HSCT |
| 16 | T0 | BASAL | Baseline Active |
| 16 | T106 | R WO TMT | Inactive post_HSCT |
| 16 | T13 | R WO TMT | Inactive post_HSCT |
| 16 | T26 | R WO TMT | Inactive post_HSCT |
| 16 | T52 | R WO TMT | Inactive post_HSCT |
| 16 | T6 | R WO TMT | Inactive post_HSCT |
| 16 | T80 | R WO TMT | Inactive post_HSCT |
| 16 | TM29 | NR WO TMT | Active post-HSCT |
| 16 | TM36 | R W TMT | Inactive post_HSCT |
| 16 | TM42 | R W TMT | Inactive post_HSCT |
| 16 | TM48 | R W TMT | Inactive post_HSCT |
| 16 | TM54 | R W TMT | Inactive post_HSCT |
| 18 | T0 | BASAL | Baseline Active |
| 18 | T13 | NR WO TMT | Active post-HSCT |
| 18 | T26 | NR W TMT | Active post-HSCT |
| 18 | T52 | R W Surgery | Inactive post_HSCT |
| 18 | TM36 | R W Surgery | Inactive post_HSCT |
| 19 | T0 | BASAL | Baseline Active |
| 19 | T106 | NR W TMT | Active post-HSCT |
| 19 | T13 | NR WO TMT | Active post-HSCT |
| 19 | T26 | NR WO TMT | Active post-HSCT |
| 19 | T52 | NR W TMT | Active post-HSCT |
| 19 | T6 | NR WO TMT | Active post-HSCT |
| 19 | T80 | NR W TMT | Active post-HSCT |
| 20 | T0 | BASAL | Baseline Active |
| 20 | T106 | R WO TMT | Inactive post_HSCT |
| 20 | T13 | R WO TMT | Inactive post_HSCT |
| 20 | T26 | R WO TMT | Inactive post_HSCT |
| 20 | T52 | R WO TMT | Inactive post_HSCT |
| 20 | T6 | R WO TMT | Inactive post_HSCT |
| 20 | T80 | R WO TMT | Inactive post_HSCT |
| 20 | TM30 | R WO TMT | Inactive post_HSCT |
| 21 | T0 | BASAL | Baseline Active |
| 21 | T13 | NR WO TMT | Active post-HSCT |
| 21 | T6 | NR WO TMT | Active post-HSCT |
| 22 | T0 | BASAL | Baseline Active |
| 22 | T106 | R WO TMT | Inactive post_HSCT |
| 22 | T13 | R WO TMT | Inactive post_HSCT |
| 22 | T26 | R WO TMT | Inactive post_HSCT |
| 22 | T52 | R WO TMT | Inactive post_HSCT |
| 22 | T6 | R WO TMT | Inactive post_HSCT |
| 22 | TM30 | R WO TMT | Inactive post_HSCT |
| 23 | TM36 | R WO TMT | Inactive post_HSCT |
| 23 | T26 | R WO TMT | Inactive post_HSCT |
| 23 | T52 | R WO TMT | Inactive post_HSCT |
| 23 | T80 | R WO TMT | Inactive post_HSCT |
| 23 | TM42 | R W TMT | Inactive post_HSCT |
| 25 | T26 | R WO TMT | Inactive post_HSCT |
| 26 | T0 | BASAL | Baseline Active |
| 26 | T106 | NR WO TMT | Active post-HSCT |
| 26 | T13 | R WO TMT | Inactive post_HSCT |
| 26 | T26 | R WO TMT | Inactive post_HSCT |
| 26 | T80 | R WO TMT | Inactive post_HSCT |
| 26 | TM30 | NR W TMT | Active post-HSCT |
| 27 | T0 | BASAL | Baseline Active |
| 27 | T13 | NR WO TMT | Active post-HSCT |
| 27 | T26 | NR WO TMT | Active post-HSCT |
| 27 | T39 | NR WO TMT | Active post-HSCT |
| 27 | T52 | NR W TMT | Active post-HSCT |
| 27 | T80 | NR W TMT | Active post-HSCT |
| 28 | T0 | BASAL | Baseline Active |
| 28 | T0 | BASAL | Baseline Active |
| 28 | T106 | R WO TMT | Inactive post_HSCT |
| 28 | T26 | R WO TMT | Inactive post_HSCT |
| 28 | T52 | R WO TMT | Inactive post_HSCT |
| 28 | T6 | R WO TMT | Inactive post_HSCT |
| 29 | T0 | BASAL | Baseline Active |
| 29 | T13 | R WO TMT | Inactive post_HSCT |
| 29 | T26 | NR WO TMT | Active post-HSCT |
| 30 | T0 | BASAL | Baseline Active |
| 30 | T13 | NR WO TMT | Active post-HSCT |
| 30 | T26 | NR WO TMT | Active post-HSCT |
| 30 | T52 | NR WO TMT | Active post-HSCT |
| 30 | T80 | R W TMT | Inactive post_HSCT |
| 31 | T0 | BASAL | Baseline Active |
| 31 | T26 | NR WO TMT | Active post-HSCT |
| 31 | T52 | NR W TMT | Active post-HSCT |
| 31 | T80 | R W TMT | Remission |
| 31 | T8 | NR WO TMT | Active post-HSCT |
| 33 | T0 | BASAL | Baseline Active |
| 33 | T13 | R WO TMT | Inactive post_HSCT |
| 33 | T26 | R WO TMT | Inactive post_HSCT |
| 34 | T0 | BASAL | Baseline Active |
| 35 | T13 | NR WO TMT | Active post-HSCT |
| 35 | T26 | NR WO TMT | Active post-HSCT |
| 35 | T52 | NR WO TMT | Active post-HSCT |
| 35 | T80 | R W TMT | Inactive post_HSCT |

**Supplementary Table S3:** Time-points and treatment status of individual patient samples

**R WO TMT** = Remission without treatment, **R W TMT**= Remission with treatment, **NR WO TMT**= No-remission without treatment, **NR W TMT**= No-remission with treatment, **T=** weeks post-HSCT, **TM=** months post-HSCT

**Supplementary Table S4:** Number of metabolite features after quality control and pre-processing steps
