# Supplementary material for: Integrated microbiota and metabolite profiles link Crohn’s disease to sulfur metabolism
Source: Nat Commun. 2020 Aug 28;11:4322. doi: 10.1038/s41467-020-17956-1 (PMC7456324; doi:10.1038/s41467-020-17956-1)
Supplement: Supplementary file 2 — Description of Additional Supplementary Files [file 41467_2020_17956_MOESM2_ESM.pdf]

Title: Supplementary Data file 1:
Description: Differentially abundant sulfated metabolites in CD patients

Title: Supplementary Data file 2:
Description: Correlation Similarity Matrix in the merged human donors and humanized datasets

Title: Supplementary Data file 3:
Description: Differentially abundant sulfated metabolites in humanized mice gut content

Title: Supplementary Data file 4:
Description: Human and Humanized fecal samples for metabolomics analysis

Title: Supplementary Data file 5:
Description: Identified metabolites and their annotation

Title: Supplementary Data file 6:
Description: Raw peak areas of metabolomics data generated during this study
