# Supplementary material for: Integrated microbiota and metabolite profiles link Crohn’s disease to sulfur metabolism
Source: Nat Commun. 2020 Aug 28;11:4322. doi: 10.1038/s41467-020-17956-1 (PMC7456324; doi:10.1038/s41467-020-17956-1)
Supplement: Supplementary file 9 — Reporting Summary [file 41467_2020_17956_MOESM9_ESM.pdf]

nature research

Corresponding author(s): Prof.Dirk Haller

Last updated by author(s): Jul 21, 2020

Reporting Summary

Nature Research wishes to improve the reproducibility of the work that we publish. This form provides structure for consistency and transparency in reporting. For further information on Nature Research policies, see Authors & Referees and the Editorial Policy Checklist .

Statistics

For all statistical analyses, confirm that the following items are present in the figure legend, table legend, main text, or Methods section.

n/a	Confirmed	
☐	☒	The exact sample size (*n*) for each experimental group/condition, given as a discrete number and unit of measurement
☐	☒	A statement on whether measurements were taken from distinct samples or whether the same sample was measured repeatedly
☐	☒	The statistical test(s) used AND whether they are one- or two-sided<br>*Only common tests should be described solely by name; describe more complex techniques in the Methods section.*
☐	☒	A description of all covariates tested
☐	☒	A description of any assumptions or corrections, such as tests of normality and adjustment for multiple comparisons
☐	☒	A full description of the statistical parameters including central tendency (e.g. means) or other basic estimates (e.g. regression coefficient) AND variation (e.g. standard deviation) or associated estimates of uncertainty (e.g. confidence intervals)
☐	☒	For null hypothesis testing, the test statistic (e.g. *F*, *t*, *r*) with confidence intervals, effect sizes, degrees of freedom and *P* value noted<br>*Give P values as exact values whenever suitable.*
☒	☐	For Bayesian analysis, information on the choice of priors and Markov chain Monte Carlo settings
☒	☐	For hierarchical and complex designs, identification of the appropriate level for tests and full reporting of outcomes
☒	☐	Estimates of effect sizes (e.g. Cohen's *d*, Pearson's *r*), indicating how they were calculated

Our web collection on statistics for biologists contains articles on many of the points above.

Software and code

Policy information about availability of computer code

Data collection	No software was used
Data analysis	GraphPad Prism (version 7.00; GraphPad Software, San Diego, CA)<br><br>16S rRNA gene sequencing analysis:<br>IMNGS (Lagkouvardos et al., 2016) http://imngs.org<br>Rhea R Pipeline for microbial analysis (Lagkouvardos et al., 2017) https://lagkouvardos.github.io/Rhea/<br>LEfSe (Segata et al., 2011) http://segatalab.cibio.unitn.it/tools/<br>RDP Classifier (Wang et al., 2007) https://rdp.cme.msu.edu/classifier/<br>SILVA database version 132 (Quast, Pruesse, Yilmaz, Gerken, Schweer, Glo, et al., 2013) https://www.arb-silva.de/<br>WEKA (Version 3.8) (Eibe et al., 2016) https://www.cs.waikato.ac.nz/<br>megahit version 1.1.3-0<br><br>Shotgun metagenomics analysis:<br>Trimmomatic version 0.36 (Bolger et al., 2014) http://www.usadellab.org/cms/<br>sortmeRNA version 2.1b<br>GHOSTKOALA (Kanehisa, Sato and Morishima, 2016) https://www.kegg.jp/ghostkoala/<br>PiCRUSt2 (Douglas et al.,2019)<br><br>Metabolomics data analysis:<br>MixOmics version 6.12.1.(Rohart et al., 2017) http://mixomics.org/<br>Analyst 1.6.2 software (Sciex, Darmstadt, Germany).<br>MS-DIAL software version 3.52

Flow Cytometry:
FlowJo v10 software   TreeStar   https://www.flowjo.com/

MicroPoint software (PreciPoint GmbH).

Tissue imaging:
ViewPoint Slide viewing software PreciPoint GmbH http://www.precipoint.com

For manuscripts utilizing custom algorithms or software that are central to the research but not yet described in published literature, software must be made available to editors/reviewers. We strongly encourage code deposition in a community repository (e.g. GitHub). See the Nature Research guidelines for submitting code & software for further information.

Data

Policy information about availability of data

All manuscripts must include a data availability statement. This statement should provide the following information, where applicable:
- Accession codes, unique identifiers, or web links for publicly available datasets
- A list of figures that have associated raw data
- A description of any restrictions on data availability

The demultiplexed reads for all 16S amplicon sequencing datasets from both patients and gnotobiotic humanized mice have been deposited to the NCBI Sequence Read Archive [http://www.ncbi.nlm.nih.gov/sra] under the accession No. PRJNA565903 [https://www.ncbi.nlm.nih.gov/bioproject/PRJNA565903/] and No. PRJNA565980 [https://www.ncbi.nlm.nih.gov/bioproject/PRJNA565980/], respectively. Metagenomics sequences generated during this study have been deposited at SRA NCBI under the accession No. PRJNA575186 [https://www.ncbi.nlm.nih.gov/bioproject/PRJNA575186/]. Full list of identified metabolite features is included as Supplementary Data file S9 of this paper.

Raw peak areas of metabolomics data generated during this study have been enclosed as additional supplementary datasets to the manuscript.

Code availability:The custom code for shotgun metagenomics data analysis is available at [https://github.com/HallerLab/Metagenomics-Metwaly-et-al.-2020]. The software packages used for metabolomics analysis are free and open source, including mixOmics methods available via [https://github.com/cran/mixOmics]

Field-specific reporting

Please select the one below that is the best fit for your research. If you are not sure, read the appropriate sections before making your selection.

☒ Life sciences   ☐ Behavioural & social sciences   ☐ Ecological, evolutionary & environmental sciences

For a reference copy of the document with all sections, see nature.com/documents/nr-reporting-summary-flat.pdf

Life sciences study design

All studies must disclose on these points even when the disclosure is negative.

Sample size

Sample sizes applicable to gnotobiotic humanized mice (Fig. 2) were chosen based on previous data of the variability of inflammation score observed in the IL10 knock-out (IL10 KO) mouse model. In total, 64 IL10 KO and 65 Wildtype mice were used in this study. For each colonization trial ,a group of 4-6 IL-10 KO mice and 5-6 wildtype mice were included. Each donor microbiota was transferred twice into ex-germ free mice (1xgavage or 3xgavage) resulting in a total of 9-12 IL10 KO or 10-12 wildtype mice colonized with each human donor microbiota.

Sample size for each experiment is indicated in the figure legends. Sample sizes for mouse experiments were determined by the current standard used and based on the minimal amount of mice required to detect significance with an alpha rate set at .05 in a standardly powered experiment.

Patient-derived human samples were collected within a longitudinal clinical cohort in Barcelona. Sample collection depended on diagnosis and patient inclusion. A number of 133 fecal samples from 29 Crohn’s disease patients undergoing hematopoietic stem cell transplant were collected during a prospective longitudinal study performed in Barcelona (Department of Gastroenterology, Hospital Clinic Barcelona) as published previously (Jauregui-Amezaga et al., 2016; López-García et al., 2017; Corraliza et al., 2019). Patient characteristics at inclusion are shown in (Table 1, Supplementary Table S1, S2 and S3).

Data exclusions

No data was excluded from the analysis. Choice of samples for each analysis type is explained in detail in the methods and results section of the manuscript

Replication

Patient-derived human samples were collected within a longitudinal clinical cohort in Barcelona. There was no attempt to replicate all aspects of sample collection. Sample collection depended on diagnosis and inclusion. Raw data is available and computational tools we used are cited

	for data reproducibility.<br><br>For mouse experiments, each experiment presented in the paper was repeated in multiple animals (between 4 and 6 per experiment). All results in the paper are drawn from the analysis of multiple animals colonized with the each human microbiota (biological replicates). Each human donor microbiota was transferred into two groups of mice using two different protocols (1xgavage and 3x gavage). Tissue analysis and microbiome profiling were performed on all mice included in the study (64 IL10 KO and 65 wildtype, in total). Experimental findings in mice were reproducible. Tissue analysis, imaging and immune phenotyping are all based on multiple animals for a given experiment.
Randomization	Patients' groups could not be randomized as they depended on diagnosis.Clinical assessments with measurement of the Crohn's Disease Activity Index and biomarkers including C reactive protein and fecal calprotectin were performed at baseline (before HSCT) and at months 3, 6, and every 6 months up to month 60. Endoscopic activity was assessed at baseline, months 6, 12, and every 12 months, and also when disease activity was suspected based on clinical and biomarker assessments, until month 60. Based on clinical and endoscopic parameters, patients were grouped as: in (active or inactive disease), or in (baseline, remission or relapse) and based on sampling time (before or after HSCT transplant).<br><br>Mouse experiments: before experiments, littermates were combined and randomly assigned to treatment groups.
Blinding	Mice were assigned randomly to groups and not blinded. Histopathological assessment of humanized mice was performed blindly, by a single observer. The investigators were not blinded during sample collection. Blinding was used during tissue analysis . Computational analysis was not performed blinded.

Reporting for specific materials, systems and methods

We require information from authors about some types of materials, experimental systems and methods used in many studies. Here, indicate whether each material, system or method listed is relevant to your study. If you are not sure if a list item applies to your research, read the appropriate section before selecting a response.

Materials & experimental systems

n/a	Involved in the study
☐	☒ Antibodies
☒	☐ Eukaryotic cell lines
☒	☐ Palaeontology
☐	☒ Animals and other organisms
☐	☒ Human research participants
☐	☒ Clinical data

Methods

n/a	Involved in the study
☒	☐ ChIP-seq
☐	☒ Flow cytometry
☒	☐ MRI-based neuroimaging

Antibodies

Antibodies used	APC-Cy7-conjugated anti-CD3 BioLegend Cat#100222<br>PE-Cy7-conjugated anti-CD4 BioLegend Cat#100528#<br>PE-conjugated anti-Foxp3 eBioscience Cat#12-5773-80<br>PerCP/Cy5.5-conjugated anti- CD62L BioLegend Cat#104432<br>APC-conjugated anti-CD44 BD Pharmingen Biosciences Cat#559250<br>PE-conjugated anti-CD8a BioLegend Cat# 100708<br>APC-conjugated anti-CD25 BioLegend Cat# 102012
Validation	All commercially available antibodies used in the study have been appropriately annotated in the manuscript (origin, species and dilutions used)

Animals and other organisms

Policy information about studies involving animals; ARRIVE guidelines recommended for reporting animal research

Laboratory animals	Germ-free wild-type male/female (WT) and IL-10-deficient (Il-10−/−) mice on 129Sv/Ev background were kept at the gnotobiology core facility of the Institute for food & health, Technical University Munich, Germany. GF mice were housed in flexible film isolators ventilated via HEPA-filtered air at 22 ± 1°C with a 12-h light/dark cycle.<br><br>Before experiments, littermates were combined and randomly assigned to treatment groups. A maximum of 5 mice are housed per cage (floor area ~540 cm2). Mice received a standard diet (autoclaved V1124-300; Ssniff, Soest, Germany) and autoclaved water ad libitum.<br><br>For fecal microbiota transplantation, male/female GF wild-type (WT) and IL-10-deficient (Il-10−/−/ SvEv129) mice (8 weeks of age) received 100 µL each of the human fecal suspension via oral gavage (one time, or three times on three consecutive days) using 20 Gauge gavage needle (Fine Science Tools).

Wild animals	The study did not involve wild animals
Field-collected samples	The study did not involve samples collected from the field
Ethics oversight	Mouse experiments and the treatment protocols were approved by the Committee on Animal Health and Care of the local government body of the state of Upper Bavaria (Regierung von Oberbayern; approval number 55.2-1-54-2532-133-2014) and performed in compliance with the EEC recommendations for the care and use of Lab. Anim. (European Communities Council Directive of 24 November 1986 (86/609/EEC))

Note that full information on the approval of the study protocol must also be provided in the manuscript.

Human research participants

Policy information about studies involving human research participants

Population characteristics	Population characteristics are presented in Table 1, Supplementary Tables S1, S2 and S3.
Recruitment	Only patient-derived fecal samples were used. Fecal samples from Crohn’s disease patients undergoing hematopoietic stem cell transplant were collected during a prospective longitudinal study performed in Barcelona (Department of Gastroenterology, Hospital Clinic Barcelona) as published previously (Jauregui-Amezaga et al., 2016; López-García et al., 2017; Corraliza et al., 2019). From this cohort, fecal samples from 29 patients with up to 5-year follow up were included. Samples were collected in the clinic or at home and subsequently delivered within 24 hours to the clinic for further processing. Homogenized samples were stored in 20% glycerol/PBS solution at −80°C. Patient-derived human samples were collected within a longitudinal clinical cohort in Barcelona. There was no attempt to replicate all aspects of sample collection. Sample collection depended on diagnosis and inclusion.
Ethics oversight	Refer to previous publications (Jauregui-Amezaga et al., 2016; López-García et al., 2017; Corraliza et al., 2019).

Note that full information on the approval of the study protocol must also be provided in the manuscript.

Clinical data

Policy information about clinical studies

All manuscripts should comply with the ICMJEguidelines for publication of clinical research and a completedCONSORT checklist must be included with all submissions.

Clinical trial registration	NCT00297193
Study protocol	Transplant study for patients with relapsing Crohn's disease demonstrating clear intolerance or toxicity to conventional treatment. The purpose of this study is to determine whether there is a potential clinical benefit of hematopoietic stem cell mobilisation followed by high dose immuno-ablation and autologous stem cell transplantation versus hematopoietic stem cell mobilisation only followed by best clinical practice.<br><br>Procedure: Autologous haematopoietic stem cell transplant. Open label, phase III, randomised, multicentre study comparing early transplantation procedure with transplantation carried out to the same protocol but delayed by one year. The status of patients undergoing early HSCT will be evaluated after one year and compared to those about to undergo delayed HSCT.<br><br>Patients were randomised to:<br>- Hematopoietic stem cell mobilisation followed, within 4 weeks, by high dose immunoablation and autologous stem cell transplantation<br>- Hematopoietic stem cell mobilisation followed, after 59 weeks, by high dose immunoablation and autologous hematopoietic stem cell transplantation<br>- All patients will be mobilised prior to randomisation. Those receiving early transplantation will be compared over the first year with those whose transplant has been delayed<br><br>[https://clinicaltrials.gov/ct2/show/NCT00297193?term=ASTIC&draw=2&rank=1]
Data collection	Patients were recruited between 2013 and 2015. Stool samples were collected at baseline and every 6 months post-HSCT treatment.
Outcomes	Primary Outcome Measures :Proportion patients in sustained disease remission [Time Frame: 1 year]. To evaluate the potential clinical benefit of hematopoietic stem cell mobilisation followed by high dose immuno-ablation and autologous stem cell transplantation versus hematopoietic stem cell mobilisation only followed by best clinical practice in patients with Crohn' s disease.<br><br>The ASTIC primary endpoint was sustained disease remission at one year, defined as complex variable comprised of three components: (1) CDAI less than 150 for at least the last 3 months (2) no active treatment during the last three months (3) no mucosal erosion or ulceration in the gastrointestinal tract.<br><br>Secondary Outcome Measures : patients who have not responded to immunosuppressant medication [Time Frame: 1 - 2 years]

To evaluate the safety of Hematopoietic Stem Cell Transplantation (HSCT) in Crohn's disease patients who have not responded to immunosuppressant medication

Other Outcome Measures:
Impact of HSCT on health related, and generic, quality of life measures [Time Frame: 1 - 2 Years]
To evaluate the impact of HSCT on health related, and generic, quality of life measures

To identify factors predictive of success [Time Frame: 1-2 years]
To identify factors predictive of success

Flow Cytometry

Plots

Confirm that:

- [x] The axis labels state the marker and fluorochrome used (e.g. CD4-FITC).
- [x] The axis scales are clearly visible. Include numbers along axes only for bottom left plot of group (a 'group' is an analysis of identical markers).
- [x] All plots are contour plots with outliers or pseudocolor plots.
- [x] A numerical value for number of cells or percentage (with statistics) is provided.

Methodology

Sample preparation	Mesenteric lymph nodes (MLNs) were harvested from humanized mice and dispersed into single-cell suspensions by homogenizing them through a 70-µm nylon cell strainer (BD Biosciences). Cells were washed and resuspended in ice-cold PBS supplemented with FCS (2%, Merck) and EDTA (1 mM, Sigma-Aldrich). FcR block was done by applying the FcR blocking reagent from Miltenyi following the manufacturer's instructions. Dead cells were excluded by applying the Zombie GreenTM Fixable Viability Kit (BioLegend). Intracellular staining was performed by using the eBioscience™ Foxp3 / Transcription Factor Staining Buffer Set (Thermo Fisher Scientific). Allophycocyanin-Cy7-conjugated anti-CD3 (17A2), PE-Cy7-conjugated anti-CD4 (RM4-5), PE-conjugated anti-CD8 (53-6.7), and PerCP/Cyanine5.5-conjugated anti-CD62L (MEL-14), Allophycocyanin-conjugated anti-CD25 (PC61.5) were from BioLegend. Allophycocyanin-conjugated anti-CD44 (IM7) was from BD Biosciences.
Instrument	FACS LSRII system (BD Biosciences)<br>Data output was analyzed by using FlowJo software.
Software	FACSDiva and FlowJo software were used to collect and analyze flow cytometry data.<br>Further data analysis was performed using the statistical software package GraphPad Prism (version 7.00; GraphPad Software, San Diego, CA).
Cell population abundance	Flow cytometry was used for quantification purposes only (no postsorting fractions were collected)
Gating strategy	For all experiments FSC-A/ SSC-A gates of the starting cell population were used to discriminate between viable cells and cell debris. Singlet and doublet cells were discriminated using FSC-A/ FSC-W gating. Isotype control stained cells were used to distinguish between background staining and specific antibody staining. Fluorescence minus one control stainings were performed.

- [x] Tick this box to confirm that a figure exemplifying the gating strategy is provided in the Supplementary Information.
